# Comparative pathogenicity of SARS-CoV-2 Omicron subvariants including BA.1, BA.2, and BA.5

**DOI:** 10.1038/s42003-023-05081-w

**Published:** 2023-07-24

**Authors:** Tomokazu Tamura, Daichi Yamasoba, Yoshitaka Oda, Jumpei Ito, Tomoko Kamasaki, Naganori Nao, Rina Hashimoto, Yoichiro Fujioka, Rigel Suzuki, Lei Wang, Hayato Ito, Yukie Kashima, Izumi Kimura, Mai Kishimoto, Masumi Tsuda, Hirofumi Sawa, Kumiko Yoshimatsu, Yuki Yamamoto, Tetsuharu Nagamoto, Jun Kanamune, Yutaka Suzuki, Yusuke Ohba, The Genotype to Phenotype Japan (G2P-Japan) Consortium, Saori Suzuki, Marie Kato, Zannatul Ferdous, Hiromi Mouri, Kenji Shishido, Naoko Misawa, Keiya Uriu, Yusuke Kosugi, Shigeru Fujita, Mai Suganami, Mika Chiba, Ryo Yoshimura, So Nakagawa, Jiaqi Wu, Akifumi Takaori-Kondo, Kotaro Shirakawa, Kayoko Nagata, Yasuhiro Kazuma, Ryosuke Nomura, Yoshihito Horisawa, Yusuke Tashiro, Yugo Kawai, Takao Hashiguchi, Tateki Suzuki, Kanako Kimura, Jiei Sasaki, Yukari Nakajima, Ayaka Sakamoto, Naoko Yasuhara, Takashi Irie, Ryoko Kawabata, Terumasa Ikeda, Hesham Nasser, Ryo Shimizu, Monira Begum, Otowa Takahashi, Kimiko Ichihara, Takamasa Ueno, Chihiro Motozono, Mako Toyoda, Akatsuki Saito, Yuri L. Tanaka, Erika P. Butlertanaka, Maya Shofa, Kaori Tabata, Isao Yokota, Keita Matsuno, Kazuo Takayama, Shinya Tanaka, Kei Sato, Takasuke Fukuhara

**Affiliations:** 1 Department of Microbiology and Immunology, Faculty of Medicine, Hokkaido University, Sapporo, Japan; 2 Institute for Vaccine Research and Development, HU-IVReD, Hokkaido University, Sapporo, Japan; 3 Division of Systems Virology, Department of Microbiology and Immunology, The Institute of Medical Science, The University of Tokyo, Tokyo, Japan; 4 Faculty of Medicine, Kobe University, Kobe, Japan; 5 Department of Cancer Pathology, Faculty of Medicine, Hokkaido University, Sapporo, Japan; 6 Department of Cell Physiology, Faculty of Medicine, Hokkaido University, Sapporo, Japan; 7 Global Station for Biosurfaces and Drug Discovery, Hokkaido University, Sapporo, Japan; 8 AMED-CREST, Japan Agency for Medical Research and Development (AMED), Tokyo, Japan; 9 Division of International Research Promotion, International Institute for Zoonosis Control, Hokkaido University, Sapporo, Japan; 10 One Health Research Center, Hokkaido University, Sapporo, Japan; 11 Center for iPS Cell Research and Application (CiRA), Kyoto University, Kyoto, Japan; 12 Institute for Chemical Reaction Design and Discovery (WPI-ICReDD), Hokkaido University, Sapporo, Japan; 13 Graduate School of Frontier Sciences, The University of Tokyo, Kashiwa, Japan; 14 Division of Molecular Pathobiology, International Institute for Zoonosis Control, Hokkaido University, Sapporo, Japan; 15 International Collaboration Unit, International Institute for Zoonosis Control, Hokkaido University, Sapporo, Japan; 16 Institute for Genetic Medicine, Hokkaido University, Sapporo, Japan; 17 HiLung Inc, Kyoto, Japan; 18 Department of Biostatistics, Faculty of Medicine, Hokkaido University, Sapporo, Japan; 19 Division of Risk Analysis and Management, International Institute for Zoonosis Control, Hokkaido University, Sapporo, Japan; 20 Graduate School of Medicine, The University of Tokyo, Tokyo, Japan; 21 Graduate School of Frontier Sciences, The University of Tokyo, Kashiwa, Chiba Japan; 22 International Research Center for Infectious Diseases, The Institute of Medical Science, The University of Tokyo, Tokyo, Japan; 23 International Vaccine Design Center, The Institute of Medical Science, The University of Tokyo, Tokyo, Japan; 24 Collaboration Unit for Infection, Joint Research Center for Human Retrovirus Infection, Kumamoto University, Kumamoto, Japan; 25 CREST, Japan Science and Technology Agency, Kawaguchi, Japan; 26 Laboratory of Virus Control, Research Institute for Microbial Diseases, Osaka University, Suita, Japan; 27 Tokai University School of Medicine, Isehara, Japan; 28 Kyoto University, Kyoto, Japan; 29 Hiroshima University, Hiroshima, Japan; 30 Kumamoto University, Kumamoto, Japan; 31 University of Miayzaki, Kumamoto, Japan; 32 Kyushu University, Kumamoto, Japan

**Keywords:** SARS-CoV-2, Pathogens, Virology

## Abstract

The unremitting emergence of severe acute respiratory syndrome coronavirus 2 (SARS-CoV-2) variants necessitates ongoing control measures. Given its rapid spread, the new Omicron subvariant BA.5 requires urgent characterization. Here, we comprehensively analyzed BA.5 with the other Omicron variants BA.1, BA.2, and ancestral B.1.1. Although in vitro growth kinetics of BA.5 was comparable among the Omicron subvariants, BA.5 was much more fusogenic than BA.1 and BA.2. Airway-on-a-chip analysis showed that, among Omicron subvariants, BA.5 had enhanced ability to disrupt the respiratory epithelial and endothelial barriers. Furthermore, in our hamster model, in vivo pathogenicity of BA.5 was slightly higher than that of the other Omicron variants and less than that of ancestral B.1.1. Notably, BA.5 gains efficient virus spread compared with BA.1 and BA.2, leading to prompt immune responses. Our findings suggest that BA.5 has low pathogenicity compared with the ancestral strain but enhanced virus spread /inflammation compared with earlier Omicron subvariants.

## Abstract (web summary)

The virological and pathological features of Omicron subvariants are compared in in vitro cellular and airway-on-a-chip and in vivo hamster models.

## Introduction

In recent months, multiple Omicron sub-lineages of severe acute respiratory syndrome coronavirus 2 (SARS-CoV-2) have emerged^1^ and raised public concern about the need for ongoing COVID-19 control measures. The subvariants named BA.4/BA.5 were first isolated in South Africa^2^, but they have now been detected in dozens of countries worldwide. In combination, they stood for over 50% of total infections in recent weeks, as of July 2022 when we launch this study. By August 2022, BA.5 had outcompeted the original BA.2 and become the dominant variant globally. The rising number of cases of COVID-19 due to BA.4/BA.5 indicates that these variants have acquired enhanced transmission ability compared with the sister lineages, BA.1 and BA.2. Indeed, as shown in a recent report from Portugal where an outbreak of BA.5 occurred^3^, the morbidity of BA.5 is higher than that of BA.1 variants. To answer public concerns whether an urgent response to this COVID-19 wave is required, several groups examined the immunoprophylactic ability against BA.5 of vaccination or infection with previous variants. Recent reports showed that BA.4 and BA.5 effectively escape from neutralizing antibodies induced by vaccination or infection^4–8^. Genome sequencing and evolutionary analyses showed that BA.4 and BA.5 are more similar to BA.2 than to the BA.1 strain, which surged in late 2021. We have shown that viral spike protein is one of the major determinants of virulence^9–12^. BA.4 and BA.5 have identical spike proteins and carry their own unique mutations, including L452R that confers enhanced fusogenic activity and resistance to the immunity induced by infection with early variants^13^. This observation is consistent with a series of our studies using recombinant viruses but with replacement of the spike protein gene from the ancestral early pandemic variants. However, the virological characteristics of the bona fide BA.5 strain isolated from COVID-19 patients have not yet been fully defined. Here, we employed the indicated Omicron subvariants (BA.1 lineage, strain TY38-873, GISAID ID: EPI_ISL_7418017; BA.2 lineage, strain TY40-385, GISAID ID: EPI_ISL_9595859; BA.5 lineage, strain TKYS14631, GISAID ID: EPI_ISL_12812500)^14^ to investigate virological characters in in vitro and in vivo models.

## Results

### Virological features of Omicron subvariants in vitro

**Fig. 1:**
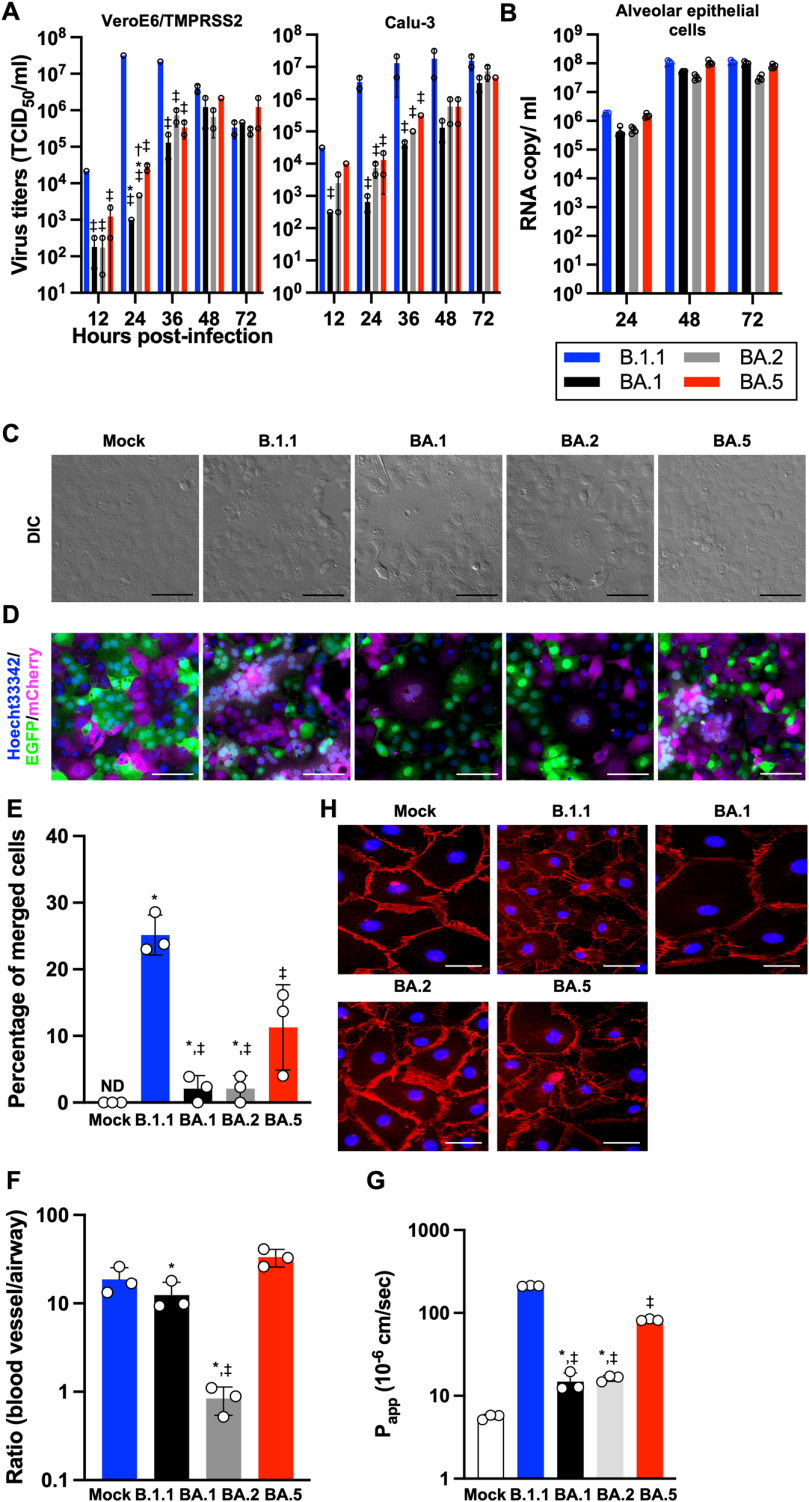
Virological features of Omicron subvariants in vitro. **A**, **B** Growth kinetics of Omicron subvariants. The four clinical isolates of B.1.1, Omicron BA.1, BA.2, and BA.5 were inoculated into VeroE6/TMPRSS2, Calu-3 (**A**), and human alveolar epithelial (**B**) cells, the infectious titers in the supernatant from VeroE6/TMPRSS2 and Calu-3 cells were determined by a TCID_50_ assay and the copy number of the viral RNA in the supernatant from human alveolar epithelial cells was quantified by RT-qPCR. **C** Bright-field images of infected VeroE6/TMPRSS2 cells (m.o.i. = 0.01) at 32 h.p.i. **D** SARS-CoV-2 induced syncytial formation. EGFP- and mCherry-expressing VeroE6/TMPRSS2 cells were co-cultured at a 1:1 ratio and infected with B.1.1, BA.1, BA.2, and BA.5 isolates. Scale bars, 200 μm. Syncytial formation was monitored by immunofluorescent microscopy at 32 h.p.i. Nuclei were counter-staining with Hoechst 33342. Scale bars, 100 μm. The representative images are shown. **E** The percentage of nuclei in the syncytia was calculated and shown as a bar graph. ND: not detected. **F** Airway-on-a-chip analysis. Medium containing SARS-CoV-2 was injected into the airway channel, which was then cultured for 6 days. Viral RNA in the supernatant of both airway and blood vessel channels was quantified by RT-qPCR. The ratio of viral invasion toward blood vessel channels was calculated (blood vessel channel/airway channel) on 6 d.p.i., as shown by percentages. **G** FD4 permeability assay of uninfected and infected airway-on-a-chip at 6 d.p.i. *P*_*app*_, apparent permeability coefficient. **H** Immunofluorescent staining for VE-cadherin (red) in HMVEC-L in the uninfected and infected airway-on-a-chip. Nuclei were counterstained with DAPI (blue). Scale bars, 10 μm. Assays were performed independently in duplicate (**A**) or triplicate (**B**, **E**, **F**, **G**). In **G**, statistically significant differences between B1.1 and other variants (^‡^*P* < 0.05), between BA.5 and other variants (*P < 0.05), and between BA.1 and other variants (^†^*P* < 0.05) were determined by Tukey’s multiplicity correction. In **E**, the statistical significance of differences between B1.1 and other variants (^‡^*P* < 0.05), and between BA.5 and other variants (*P < 0.05) were determined by Tukey’s multiplicity correction. In **F**, using Tukey’s multiplicity correction test, the ratio (blood vessel channel/airway channel) of BA.5 was significantly higher than that of BA.1 and BA.2. Both the ratio of B.1.1 and BA.1 was also significantly higher than that of BA.2. In **G**, statistically significant differences between B1.1 and other variants (^‡^*P* < 0.05), and between BA.5 and other variants (*P < 0.05) were determined by Tukey’s multiplicity correction.

It is well documented that the S protein is one of the major virulence determinants. The newly emerged Omicron subvariant BA.5 was a descendant of BA.2, and these two subvariants share most of the S substitutions (Supplementary Fig. 1)^13^. Compared with BA.2, BA.5 harbors five substitutions: L452R, HV69-70del, and F486V, and a revertant R493Q (Supplementary Fig. 1). First, to elucidate the virological characteristics of these clinical isolates, we obtained BA.1 isolate (strain TY38-873), BA.2 isolate (strain TY40-385), and BA.5 isolate (strain TKYS14631). A D614G-bearing early-pandemic B.1.1 isolate (strain TKYE610670)^10^ was used as a control. We characterized the isolates’ in vitro growth kinetics using the cell lines VeroE6/TMPRSS2^15^, Calu-3, and iPS cell-derived alveolar epithelial cells (Fig. 1A, B, and Supplementary Fig. 2A). In the cell lines VeroE6/TMPRSS2 and Calu-3, B.1.1 exhibited relatively high replication efficiency compared to the three Omicron subvariants. Among the Omicron subvariants, BA.1 showed a low replication rate compared to the later subvariants, BA.2 and BA.5. In the iPS cell-derived alveolar epithelial cells, B.1.1, BA.1, and BA.5 exhibited slightly high replication efficiency compared with strain BA.2, suggesting that the replication of Omicron subvariant BA.5 in vitro is similar to the ancestral variants. Because in vitro viral fusogenicity is one of the indicators for pathogenicity^12^, we investigated the fusogenic characters of the Omicron subvariants. In the infected cells, they exhibited quite different morphologies; B.1.1 formed larger syncytia than the Omicron subvariants (Fig. 1C, and Supplementary Fig. 2B). To quantify syncytial formation of the Omicron subvariants, we generated VeroE6/TMPRSS2 cells expressing either EGFP or mCherry and seeded equal amounts of them for syncytial formation. When the respective colored cells form syncytia, a color turned to be a merged image (Fig. 1D and Supplementary Fig. 2C). Among the Omicron subvariants, the ability of BA.1 and BA.2 to form syncytia was significantly lower than that of BA.5 (Fig. 1E and Supplementary Fig. 2D). Consistent with the observation of syncytia, B.1.1 showed efficient cleavage of S protein (Supplementary Fig. 2E). These findings suggest that, even though Omicron subvariants are still less fusogenic than the B.1.1 isolate, the subvariants are evolving toward efficient fusogenicity in VeroE6/TMPRSS2 cells. Next, to evaluate the influence of viral infection on the respiratory epithelial and endothelial barriers, airway-on-a-chip was used; by examining the amount of virus that migrates from the airway channel to the blood vessel channel, the ability to disrupt the respiratory epithelial and endothelial barriers can be evaluated^16^. Airway/lung-on-a-chips are known to be useful tools for analyzing the behavior of pathogens including SARS-CoV-2^17–19^. Disruptions of the respiratory epithelial and endothelial barriers can be observed in Airway/lung-on-a-chips infected with SARS-CoV-2 (Fig. 1F and Supplementary Fig. 2F). In the Omicron subvariants, the largest amount of virus was detected in the blood vessel channel of BA.5-infected airway-on-a-chip compared with BA.1 and BA.2 at 6 days post-infection (d.p.i.) (Supplementary Fig. 2F). In addition, the B.1.1- and BA.5-infected airways-on-a-chip exhibited more severe disruption than the BA.1- and BA.2-infected ones (Fig. 1G and 1H), suggesting that BA.5 possesses substantial respiratory epithelial and endothelial barrier disruption capacity. Consistent with this observation, the expression level of CLDN5, a component necessary for sustaining the vascular endothelial barrier, was observed to be decreased in endothelial cells of B.1.1- and BA.5-infected airways-on-a-chip (Supplementary Fig. 2G). Of note, the SARS-CoV-2 infection-mediated barrier disruption may be caused not only by disruption of the vascular endothelial barrier but also by damage to the airway epithelial cell layer. Furthermore, airways-on-a-chip cannot perfectly reproduce in vivo conditions (e.g. endothelial robustness), thus making it imperative to conduct further validation utilizing animal models.

### Virological features of Omicron subvariants in vivo

**Fig. 2:**
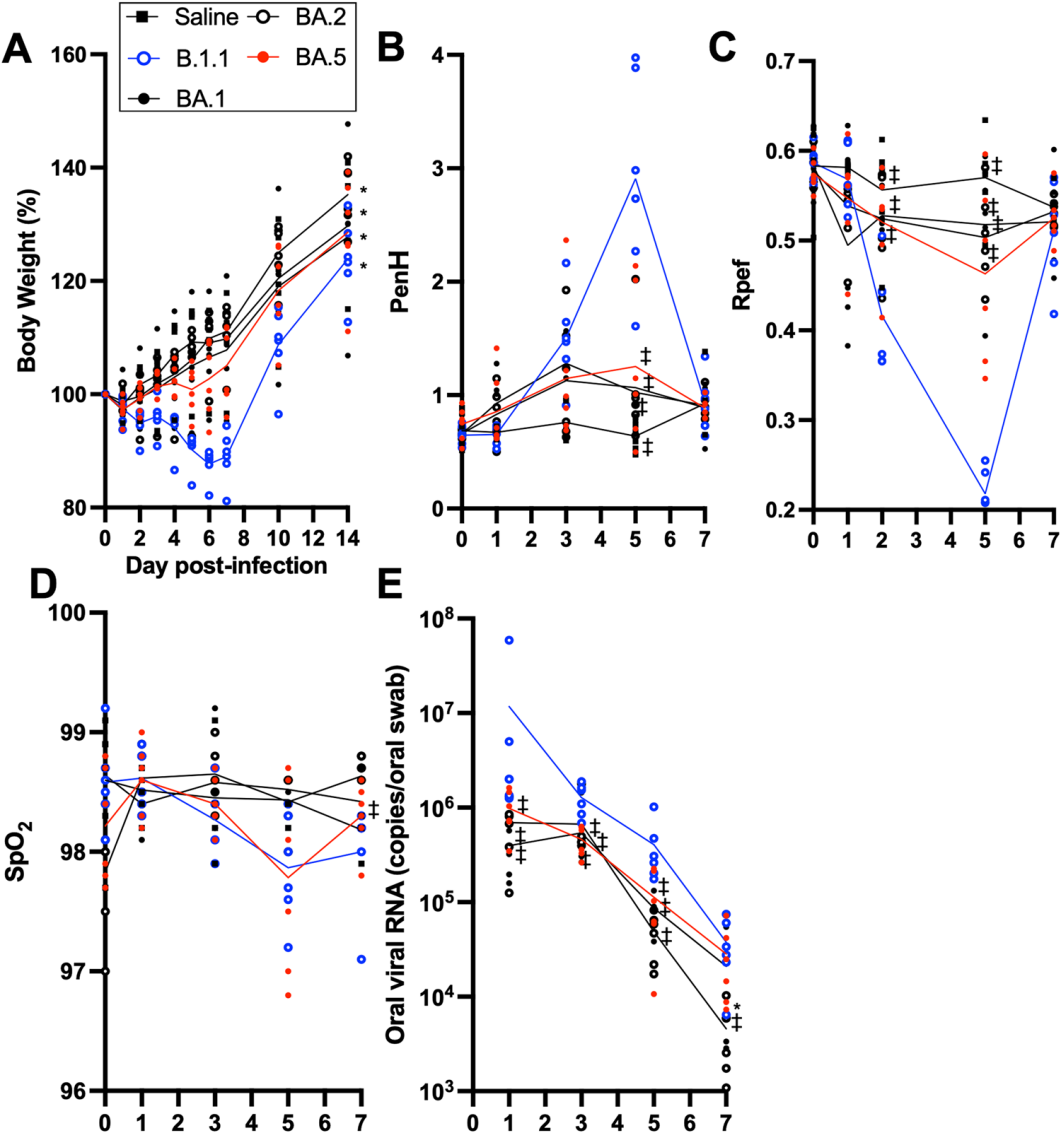
Time-course dynamics of Omicron subvariants in vivo. Syrian hamsters were intranasally inoculated with saline (*n* = 6, uninfected control), B.1.1 (*n* = 6), BA.1 (*n* = 5), BA.2 (*n* = 6), and BA.5 (*n* = 6). Body weight (**A**), PenH (**B**), Rpef (**C**), SpO_2_ (**D**), and viral RNA load in the oral swab (**E**) were routinely measured as indicated in the graph. Data are the mean ± s.e.m. In **A**, the statistical significance of differences between BA.5 and other variants or saline across timepoints from 1 d.p.i. to 7 d.p.i. was determined by multiple regression (*P < 0.05). The family-wise error rates calculated using the Holm method are indicated in the figure. In **B**–**E**, the statistical significance of differences between B.1.1 and other variants or saline was tested by Tukey’s multiplicity correction (^‡^*P* < 0.05). The statistical significance of differences between BA.5 and other variants or saline was tested by Tukey’s multiplicity correction (*P < 0.05).

To investigate the dynamics of viral replication in vivo and the pathogenicity of Omicron subvariants, we used the established animal model using hamsters^9–11^. Consistent with our previous study, B.1.1-infected hamsters exhibited decreased body weight from 2 d.p.i. (Fig. 2A). The change of the body weight of Omicron subvariant-infected hamsters was moderate compared with that of B.1.1-infected hamsters and similar to that of the uninfected group. Notably, the dynamics of weight changes of BA.5-infected hamsters were significantly different from those of the BA.2-infected and uninfected hamsters (Fig. 2A and Supplementary Fig. 3A). We then quantitatively analyzed the pulmonary function of infected hamsters as reflected by three parameters: enhanced pause (PenH) and the ratio of time to peak expiratory flow relative to the total expiratory time (Rpef), which are surrogate markers for bronchoconstriction or airway obstruction, and subcutaneous oxygen saturation (SpO_2_). As shown in Fig. 2B–D and Supplementary Fig. 3B–D, the B.1.1-infected hamsters exhibited respiratory disorders according to these three parameters. In contrast, in BA.1-, BA.2-, and BA.5-infected hamsters, the PenH value was significantly lower than those in B.1.1-infected hamsters (Fig. 2B), and the Rpef value was significantly higher than those in B.1.1-infected hamsters (Fig. 2C). As for SpO_2_ values, B.1.1- and BA.5-infected hamsters exhibited a tendency for lower levels than BA.1- and BA.2-infected hamsters but there were no significant results in pairwise comparison between subvariants in each day (Fig. 2D). In vivo viral dynamics was analyzed by collecting an oral swab of infected hamsters at the indicated timepoints (Fig. 2E). At 3 d.p.i. and 5 d.p.i., the viral loads of Omicron subvariant-infected hamsters were significantly lower than those of B.1.1-infected hamsters.

**Fig. 3:**
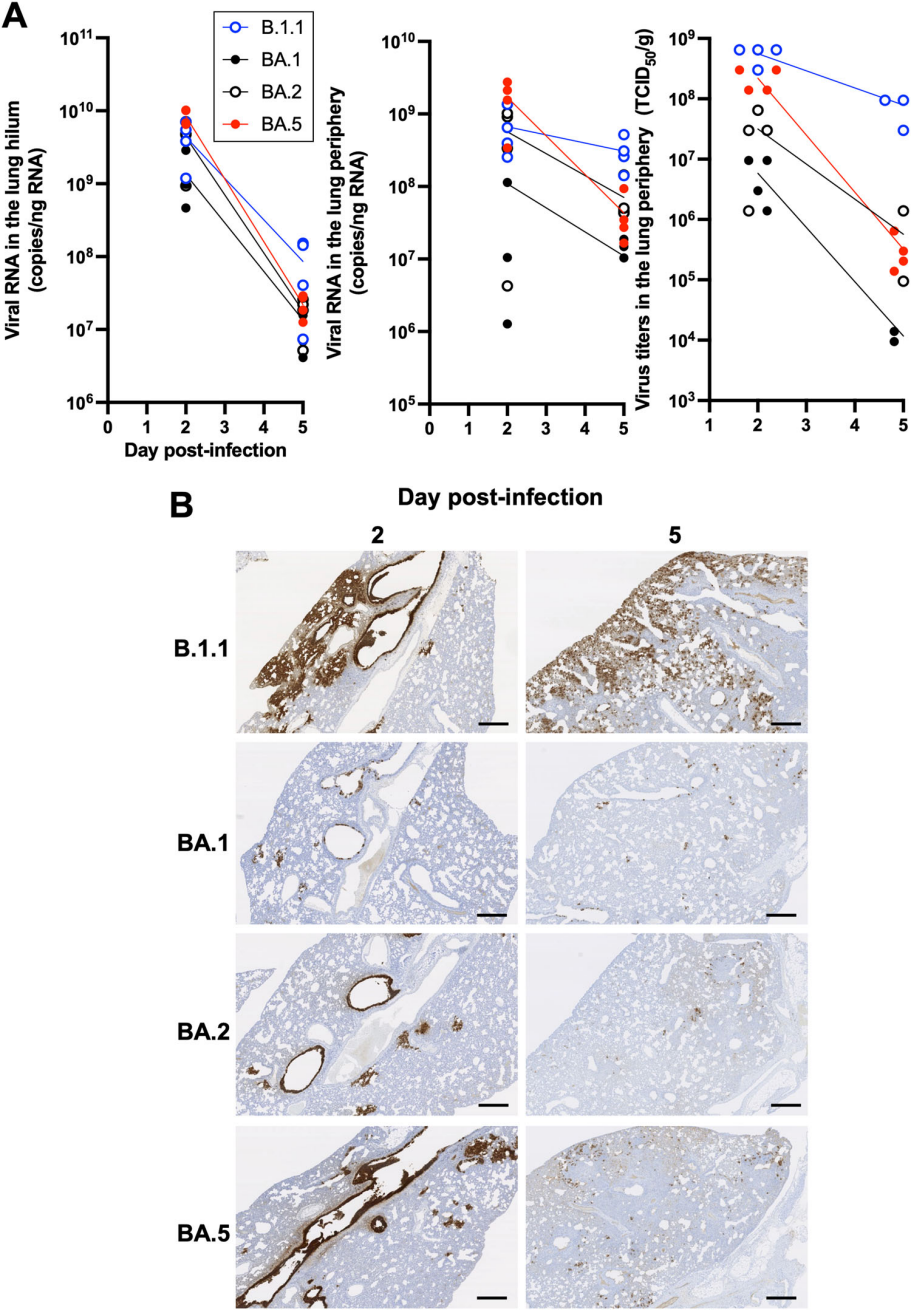
Virological features of Omicron subvariants in vivo. Syrian hamsters were intranasally inoculated with B.1.1 (*n* = 4), BA.1 (*n* = 4), BA.2 (*n* = 4), and BA.5 (*n* = 4). **A** Viral RNA quantification and titration. Viral RNA load (left, middle) in the lung hilum and periphery and viral titer (right) in the lung periphery were quantified. **B** IHC of the SARS-CoV-2 N protein in the lungs of infected hamsters. Representative IHC panels of the viral N proteins in the lower lobe of lungs of the infected hamsters. The raw data at 2 d.p.i. are shown in Supplementary Fig. 5A. Scale bars, 500 µm. In **A**, viral RNA copies and titers at each day were compared using Tukey’s multiplicity correction. As for viral RNA copies in the lung hilum area, BA.5 had significant higher copies than BA.1 at 2 d.p.i. Of viral RNA copies in the lung periphery area, BA.5 had significant higher copies than BA.1 at 2 d.p.i. B.1.1 had significantly higher copies than BA1, BA2, and BA.5 at 5 d.p.i. As for viral titers in the lung periphery area, B.1.1 had significantly higher titers than BA1, BA2, and BA.5 at 2 d.p.i. B.1.1 had significantly higher titers than BA.1 and BA.2, and BA.5 at 5 d.p.i.

We next assessed viral spread in the respiratory tissues and thus collected the trachea and lung at 2 and 5 d.p.i. (Fig. 3A). In the upper trachea of infected hamsters, epithelial cells were sporadically positive for viral N protein at 2 d.p.i., but no significant differences were found among B.1.1 and Omicron subvariants (Supplementary Fig. 4A–C). As for the lung, we investigated the viral spread in separate regions, the hilum and periphery. In the lung hilum, although viral RNA copies (Fig. 3A, left) in the lung hilum of BA.1 were ~10-fold lower than those of B.1.1, BA.2, and BA.5 at 2 d.p.i., the levels of viral RNAs of all Omicron subvariants were significantly lower than those of B.1.1 at 5 d.p.i. In contrast to the hilum, viral RNA copies (Fig. 3A, middle) and titers (Fig. 3A, right) in the periphery were slightly different among Omicron subvariants. Large amounts of viral load were detected from BA.5 at 2 d.p.i., which was comparable to B.1.1. To further characterize virus spread by Omicron subvariants, immunohistochemical (IHC) analysis of viral N protein was conducted using specimens from the respiratory system. At 2 d.p.i., the N protein was observed in the alveolar space around the bronchi/bronchioles in the B.1.1-infected hamsters (Fig. 3B, top panel, Supplementary Fig. 5A). In Omicron BA.2- and BA.5-infected hamsters, the N protein was observed in the alveolar space to a lesser extent than for B.1.1. The N proteins were strongly retained in lobar bronchi in the BA.5-, but not BA.2-, infected hamsters (Fig. 3B, BA.5: bottom panel, BA.2: third panel, Supplementary Fig. 5B). In contrast, little N protein was detected in the BA.1-infected lungs (Fig. 3B, second top panel, Supplementary Fig. 5A and Supplementary Fig. 5C). At 5 d.p.i., B.1.1 and BA.5 N proteins were also distributed in the peripheral alveolar space and the largest amount was observed in the B.1.1 group (Fig. 3B). The N proteins were hardly detected in the lungs infected with BA.1 and BA.2. These findings suggest that BA.5 efficiently infects bronchial/bronchiolar epithelium and invades alveolar space more than BA.1 and BA.2. In contrast, BA.1 and BA.2 infect only a portion of bronchial/bronchiolar epithelium and are less efficiently transmitted to the neighboring epithelial cells. Overall, the IHC data suggest that BA.5 has a higher spread of infection from the bronchi/bronchioles to the peripheral alveoli than BA.1 and BA.2, but does not reach the level of B.1.1.

### Inflammation in lung tissue infected with Omicron subvariants

**Fig. 4:**
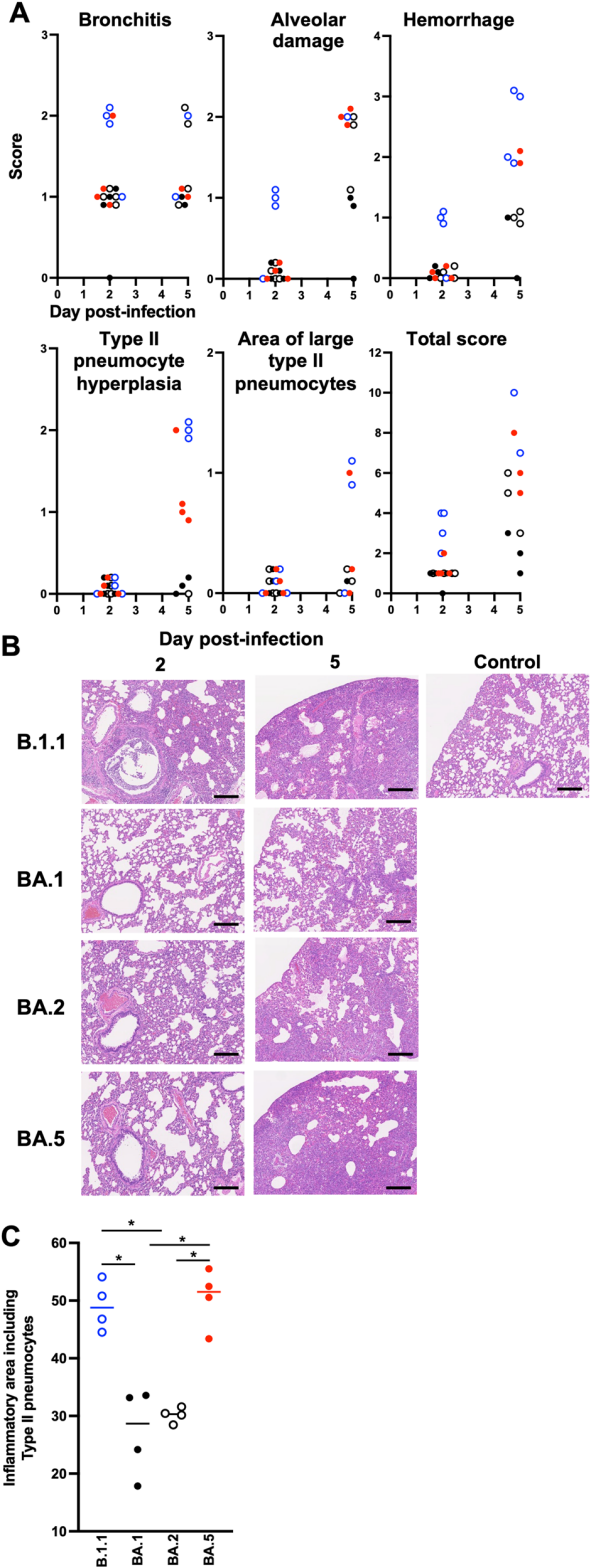
Pathological features of Omicron subvariants. Syrian hamsters were intranasally inoculated with B.1.1 (*n* = 4), BA.1 (*n* = 4), BA.2 (*n* = 4), and BA.5 (*n* = 4). **A** Histopathological scoring of lung lesions. **B** H&E staining of the lungs of infected hamsters. Uninfected lung alveolar space and bronchioles are also shown. Scale bars, 200 μm. **C** Summary of the percentage of the section represented by the inflammatory area with type II pneumocytes at 5 d.p.i. The raw data are shown in Supplementary Fig. 6A. In **C**, data are the mean ± s.e.m. and each dot indicates the result from an individual hamster. In **A**, the total score at each day was compared using Tukey’s multiplicity correction. At 2 d.p.i, B.1.1 had significantly higher score than BA1, BA2, and BA.5. At 5 d.p.i., B.1.1 had significantly higher score than BA.1 and BA.2, and BA.5 had significantly higher score than BA.1. In **C**, the statistical significance of differences (*P < 0.05) was determined by the analysis of variance using Tukey’s multiplicity correction.

To investigate the pathogenicity of Omicron subvariants in the lung, the formalin-fixed right lungs of infected hamsters were analyzed by carefully identifying the four lobules and main bronchus and lobar bronchi sectioning each lobe along with the bronchial branches. Histopathological scoring was performed as described in previous reports^9–11^ with minor modifications. Briefly, pathological features, including bronchitis or bronchiolitis, hemorrhage or congestion, alveolar damage with epithelial apoptosis and macrophage infiltration, and type II pneumocytes hyperplasia, were evaluated by certified pathologists in the second most severe pulmonary lobe with an efficient number of samples for average evaluation. The severity of these pathological findings was scored using a four-tiered system as follows: 0 (negative), 1 (weak), 2 (moderate), and 3 (severe). Bronchitis is an inflammatory indicator at an early stage of infection. At 2 d.p.i., the B.1.1-infected hamsters showed the most severe features compared with the hamsters infected with Omicron variants (Fig. 4A and 4B). At 5 d.p.i., the B.1.1-infected hamsters exhibited more severe damages than the animals infected with Omicron variants in total (Fig. 4A and 4B). Among the Omicron subvariants at 5 d.p.i, BA.5 exhibited relatively severe inflammation compared with BA.1, including hemorrhage, alveolar damage from the infiltration of lymphocytes and macrophages (score 2.0) and the presence of type II pneumocytes with enlarged cellular cytoplasm and nucleus (score 2.0) (Fig. 4A). Next, inflammatory areas mainly composed of type II pneumocytes with various inflammatory cells, such as neutrophils, lymphocytes, and macrophages (termed the area of type II pneumocytes), were morphometrically analyzed. The area of type II pneumocytes was found to be significantly larger in BA.5 (50.5%) than in BA.1 (27.2%) or BA.2 (30.2%) (Fig. 4C, Supplementary Fig. 6A, and Supplementary Fig. 6B). Area of all pathological phenotypes were mapped in Supplementary Fig. 7. Taken together, histopathological analyses indicate that BA.5 caused severe inflammation among Omicron subvariants but not reach to B.1.1.

**Fig. 5:**
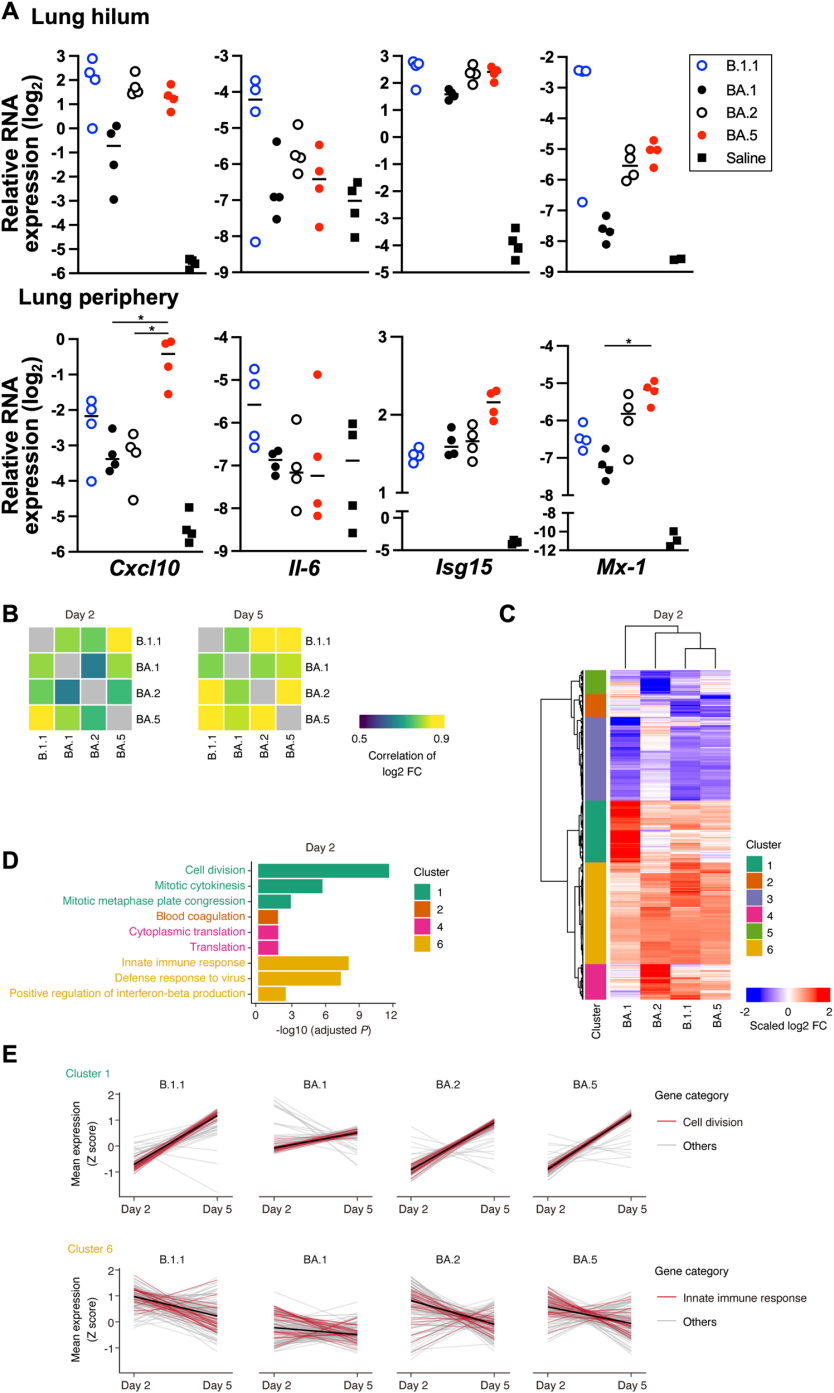
Gene expression alteration of hamster lungs upon infection with Omicron subvariants. **A** mRNA of the lung tissues obtained at day 2 post-infection was used to measure expression levels of inflammatory genes (*Cxcl10*, *Il-6*, *Isg15*, and *Mx-1*) with normalization using the housekeeping gene *Rpl18*. **B** Similarity of gene expression alteration upon infection with the Omicron subvariants and the ancestral B.1.1 lineage. Spearman’s correlation of the log 2-fold change (FC) value [infected versus uninfected hamsters] was calculated between pairs of variants. Results for 2 d.p.i. (left) and 5 d.p.i. (right) are shown. **C** Heatmap showing gene expression alteration caused by the respective variants at 2 d.p.i. The scaled fold change (FC) values for 438 genes that were differentially expressed between uninfected and infected hamsters and among the variants are shown. Genes were categorized into six clusters. Information on the clusters is summarized in Supplementary Data 1. **D** GO enrichment analysis for genes in the clusters. Of the significantly enriched GO terms, the top 3 terms are shown. The full list of GO terms is summarized in Supplementary Data 2. **E** Expression dynamics of genes in clusters 1 and 6 from 2 d.p.i. to 5 d.p.i. Gene expression levels were normalized as Z scores, and the mean values among replicates are shown. The black line indicates the mean dynamics. Genes associated with the GO terms “cell division” (for cluster 1) and “innate immune response” (for cluster 6) are colored. In **A**, each dot indicates the result from an individual hamster. The statistical significance of differences among Omicron subvariants was tested by Tukey’s multiplicity correction (*P < 0.05).

To further investigate inflammatory responses upon infection with Omicron subvariants in vivo, the mRNA of the lung hilum and periphery areas at 2 d.p.i. and modulation of four host genes (*Cxcl10*, *Il-6*, *Isg15*, and *Mx-1*) were evaluated (Fig. 5A and Supplementary Fig. 8). Upon infection with all variants, the evaluated ISGs, *Cxcl10*, *Isg15*, and *Mx-1*, were upregulated and the expression levels for B.1.1 were the highest, followed by those for BA.2 and BA.5 in the lung hilum area. The expression of *Il-6* was also upregulated and remained only in B.1.1-infected hamsters, but not in Omicron subvariant-infected ones. These findings indicate that the inflammation in the lung hilum area is similar among the Omicron subvariants. In contrast, BA.5 infection upregulated *Cxcl10*, *Isg15*, and *Mx-1* expression more than the other Omicron subvariants infection in the lung periphery area, suggesting that BA.5 might provoke severe inflammation, supporting the histopathological data.

We conducted RNA-seq analysis for investigation of the host gene expression associated with the Omicron subvariant infection in the lung hilum area. Basically, the patterns of gene expression alterations were similar among the variants, particularly at 5 d.p.i. (Fig. 5B). At 2 d.p.i., notably, alteration of gene expression upon infection with BA.5 was more similar to that with B.1.1 than that with other Omicron subvariants (Fig. 5B). This suggests that BA.5 mirrors the gene expression of B.1.1, the ancestral lineage of Omicron, rather than the parental Omicron subvariants. To characterize differences in the alteration pattern of gene expression among the Omicron subvariants at 2 d.p.i., we first extracted differentially expressed genes (DEGs) in the two-group comparison between infected and uninfected hamsters for respective variants (2,257 genes). Of these, we further extracted DEGs in the multiple group comparison among the variants (438 genes). Then, we performed hierarchical clustering analysis and Gene Ontology (GO) enrichment analysis to further characterize these associated 438 genes (Fig. 5C, D). We identified the genes upregulated specifically in BA.1 at 2 d.p.i. (cluster 1), which are enriched in genes related to cell division (Fig. 5C, D). This suggests that viral infection triggers cell proliferation, potentially for tissue repair, at the acute phase of infection (2 d.p.i.) in hamsters infected with BA.1, which exhibits lower pathogenicity in the lungs. In the variants other than BA.1, cluster 1 genes were induced on 5 d.p.i., suggesting that such cell proliferation in hamster lungs infected with these variants starts later than that with BA.1 (Fig. 5E). Furthermore, we identified genes more strongly induced by BA.2, BA.5, and B.1.1 than by BA.1 (cluster 5), which are related to innate immunity (Fig. 5C and 5D). This result suggests that variants capable of more efficient replication in hamster lungs (e.g., BA.2, BA.5, and B.1.1) cause stronger innate immune responses in lung tissues. In variants other than BA.1, the expression of cluster 5 genes also decreased belatedly on 5 d.p.i. (Fig. 5D). Moreover, we identified genes downregulated specifically in BA.5 and B.1.1 (cluster 2), associated with blood coagulation (Fig. 5B and 5C). Altogether, these findings indicate that BA.5 infection resulted in extensive inflammation throughout the lung and a disordered respiratory system, leading to increased pathogenicity compared with that of the parental Omicron subvariants.

## Discussion

Emergence of SARS-CoV-2 variants impose continuous implementation of control measurement. As of July 2022, BA.5 subvariant emerged and had been dramatically surging and outcompeting the parental BA.1 and BA.2 variants. Researchers rapidly established studies that showed further resistance of the SARS-CoV-2 Omicron variant against the immunity elicited by previous infections and vaccination^20–25^. In addition, the morbidity of COVID-19 is elevated upon infection with BA.5^3^, suggesting an urgent need for the appropriate implementation of control measures. However, comparative analyses of these Omicron subvariants—BA.1, BA.2, and BA.5—have not been well documented. Uraki et al. showed that the clinical isolate BA.5 exhibited lower pathogenicity than the ancestral Delta in hamster models^26^. We recently reported that, in our hamster model, the spike protein of BA.5 contributes to enhanced pathogenicity compared with that of the previous Omicron subvariant BA.2^13^. Here, we further investigated the in vitro and in vivo characteristics of three clinical isolates of Omicron subvariants: BA.1, BA.2, and BA.5. Although the virulence of the Omicron subvariants was lower than that of the ancestral lineage B.1.1, our comprehensive analyses suggested that BA.5 causes greater tissue inflammation/is slightly more pathogenic by evolving to enhance the inflammatory response compared with the ancestral Omicron subvariants. This might be a key factor behind the deterioration of health outcomes in humans with BA.5 infection^3^.

As we showed in a series of studies using recombinant viruses^9–12^, fusogenicity by the viral spike protein has a great impact on viral replication and pathogenesis. Consistent with this, the fusogenicity of the Omicron subvariants was lower than that of the conventional B.1.1 strain and BA.5 exhibited significant high fusogenicity among Omicron subvariants (Fig. 1C–E and Supplementary Fig. 2B–D). In addition, in vitro growth kinetics was shown to be similar in the cell lines VeroE6/TMPRSS2, Calu-3 cells, and in iPS cell-derived lung epithelial cells (Fig. 1A, B, and Supplementary Fig. 2A). However, interestingly, investigation with airway-on-a-chip that much more strongly mimics the in vivo environment was conducted, with the results showing that BA.5 has strong barrier disruption capacity among the Omicron subvariants (Fig. 1F–H, and Supplementary Fig. 2G). In our hamster models, 1 day after inoculation is a peak of viral detection^9–11,13^. Infection with BA.5 showed strong hemorrhage and alveolar damage compared with the other Omicron subvariants (Fig. 4A and 4B), supporting the assertion that the phenotype of BA.5 promotes the invasion of respiratory tissues.

Uraki et al. recently showed that Omicron subvariants, including BA.5, are less pathogenic than Delta and that the weight loss by BA.5 infection is slightly greater than that by BA.2 infection^26^. Consistent with this, in the present study, the weight loss in BA.5-infected hamsters was greater than that for other Omicron subvariants (Fig. 2A). In addition, in our previous study^13^ by using recombinant virus with replacement of the spike protein gene with the BA.2 backbone, the recombinant virus-bearing BA.4/5 spike protein exhibited significantly enhanced pathogenicity in hamsters. Taking our present and previous studies together, there is a need to underscore the possibility that the BA.5 spike protein evolved to achieve higher pathogenicity. In addition to viral characters of replication and pathogenicity, transmissibility of the Omicron subvariants should be experimentally investigated in future studies.

Although infection with the Omicron subvariants resulted in low pathogenicity in the hamster model, BA.5 exhibited slightly more exacerbated weight loss and one of the respiratory markers Rpef (Fig. 2A, B, Supplementary Fig. 3A, and Supplementary Fig. 3C) among the Omicron subvariants. BA.5 efficiently infected bronchial/bronchiolar epithelium and invaded the alveolar space, leading to replication of the remaining virus in the lungs (Fig. 3B). As shown in a clinical report, BA.5 exhibits higher morbidity than BA.2, suggesting that inflammation influences the resulting clinical manifestations. Thus, we evaluated the inflammatory responses evoked by viral infection. First, we investigated inflammation pathologically. Infection with BA.5 resulted in severe alveolar damage from the infiltration of lymphocytes and macrophages, abnormal type II pneumocytes, and a higher degree of hemorrhage compared with the parental BA.2 infection (Fig. 4A, and Supplementary Figs. 6, 7). As modulation of host genes by the Omicron subvariants infection in the lung periphery area, upregulation of *Cxcl10*, *Isg15*, and *Mx-10* expression was observed in the BA.5-infected hamsters (Fig. 5A). RNA-seq analysis revealed that the change in gene expression by BA.5 infection is similar to that of ancestral B.1.1 at the early stage of infection (Fig. 5B). By morphometric analysis that is sensitive to subtle differences in inflammation, the inflammatory area of the lungs infected with BA.5 was shown to be larger than that of BA.2 and approximately equal to that of B.1.1. at 5 d.p.i. (Fig. 4C), indicating that BA.5 is more immunopathogenic than BA.2. Reuschl et al. showed the enhanced suppression of innate immunity by BA.4 and BA.5 compared with that of the previous BA.2 subvariants^27^. Taking these findings together, the severe inflammation associated with BA.5 due to efficient viral spread in the lung reflects the severe clinical manifestations currently experienced in the human population^3^.

In summary, our analyses using clinically isolated Omicron subvariants—BA.1, BA.2, and BA.5—based on in vitro experiments and established animal models should deepen our understanding of the ecology and evolution of SARS-CoV-2. Our findings should aid the establishment of measures for efficient controlling COVID-19.

## Methods

### Ethics statement

All experiments with hamsters were performed in accordance with the Science Council of Japan’s Guidelines for the Proper Conduct of Animal Experiments. The protocols were approved by the Institutional Animal Care and Use Committee of National University Corporation Hokkaido University (approval numbers 20-0123 and 20-0060).

### Cell culture

HEK293-ACE2/TMPRSS2 cells [HEK293 cells (ATCC CRL-1573) stably expressing human ACE2 and TMPRSS2]^12^ were maintained in Dulbecco’s modified Eagle’s medium (DMEM) (high glucose) (Nacalai Tesque, Cat# 08459-64) containing 10% fetal bovine serum (FBS) and 1% penicillin-streptomycin (PS). VeroE6/TMPRSS2 cells (VeroE6 cells stably expressing human TMPRSS2; JCRB1819)^15^ were maintained in DMEM (low glucose) (Sigma-Aldrich, Cat# D6046-500ML) containing 10% FBS, G418 (1 mg/ml; Nacalai Tesque, Cat# G8168-10ML), and 1% PS. Calu-3 cells (human airway epithelial cells; ATCC HTB-55) were maintained in EMEM (Wako, Cat# 055-08975) containing 10% FBS and 1% PS. Human alveolar epithelial cells derived from human induced pluripotent stem cells (iPSCs) were manufactured in accordance with established protocols as described below (see “Preparation of human alveolar epithelial cells from human iPSCs” section) and provided by HiLung Inc.

### Preparation of human alveolar epithelial cells from human iPSCs

The air–liquid interface cultured alveolar epithelial cells were differentiated from human iPSC-derived lung progenitor cells as previously described^28–30^. Briefly, lung progenitor cells were induced in a stepwise manner from human iPSCs with reference to a 21-day, four-step protocol^28^. On day 21, lung progenitor cells were isolated with specific surface antigen carboxypeptidase M and seeded onto the upper chamber of a 24-well Cell Culture Insert (Falcon, #353104), followed by 28-day and 7-day differentiation of alveolar epithelial cells. Alveolar differentiation medium supplemented with dexamethasone (Sigma-Aldrich, Cat# D4902), KGF (PeproTech, Cat# 100-19), 8-Br-cAMP (Biolog, Cat# B007), 3-isobutyl 1-methylxanthine (IBMX), CHIR99021 (Axon Medchem, Cat# 1386), and SB431542 (FUJIFILM Wako, Cat# 198-16543) was used for the induction of alveolar epithelial cells.

### SARS-CoV-2 preparation and titration

Omicron subvariants (BA.1 lineage, strain TY38-873, GISAID ID: EPI_ISL_7418017; BA.2 lineage, strain TY40-385, GISAID ID: EPI_ISL_9595859; BA.5 lineage, strain TKYS14631, GISAID ID: EPI_ISL_12812500)^14^ were obtained from the National Institute of Infectious Diseases (BA.1 and BA.2) and Tokyo Metropolitan Institute of Public Health, Japan. An early-pandemic D614G-bearing isolate (B.1.1 lineage, strain TKYE610670; GISAID ID: EPI_ISL_479681) was used in our previous study^10^.

Virus preparation and titration were performed as previously described^10,12^. To prepare the working virus stock, 20 μl of the seed virus was inoculated into VeroE6/TMPRSS2 cells (5 × 10^6^ cells in a T-75 flask). One hour after infection, the culture medium was replaced with DMEM (low glucose) (Wako, Cat# 041-29775) containing 2% FBS and 1% PS. At 3 d.p.i., the culture medium was harvested and centrifuged, and the supernatants were collected as the working virus stock. The viral genome sequences of working viruses were verified as described below.

The titer of the prepared working virus was measured as the 50% tissue culture infectious dose (TCID_50_). Briefly, 1 day before infection, VeroE6/TMPRSS2 cells (10,000 cells) were seeded into a 96-well plate. Serially diluted virus stocks were inoculated into the cells and incubated at 37 °C for 4 days. The cells were observed under a microscope to judge the appearance of CPE. The value of TCID_50_/ml was calculated using the Reed–Muench method^31^.

### SARS-CoV-2 infection

One day before infection, VeroE6/TMPRSS2 cells and Calu-3 cells were seeded into a 12-well plate. SARS-CoV-2 was inoculated at m.o.i. = 0.1 and incubated at 37 °C for 1 h. The infected cells were washed, and 1 ml of culture medium was added. The culture supernatant (100 µl) and cells were collected at the indicated time points. The samples were subjected to analyze viral titer shown as TCID_50_ and viral RNA copy number quantified by RT-qPCR (see below). To monitor the syncytial formation in infected cell culture, bright-field photos were obtained using an Eclipse Ts2 microscope (Nikon).

The infection experiment using human iPSC-derived alveolar epithelial cells was performed as previously described^10^. Briefly, the working viruses were diluted with Opti-MEM (Thermo Fisher Scientific, Cat# 11058021). Infection experiments were done with cells at an air-liquid interface. The diluted viruses (1000 TCID_50_ in 100 μl) were inoculated onto the apical side of the culture and incubated at 37 °C for 1 h. The inoculated viruses were removed and washed twice with Opti-MEM. To harvest the viruses on the apical side of the culture, 100 μl of Opti-MEM was applied onto the apical side of the culture and incubated at 37 °C for 10 min. The Opti-MEM applied was harvested and used for RT-qPCR to quantify the viral RNA copy number (see below).

### Syncytial formation assay

An equal number of VeroE6/TMPRSS2 cells expressing either EGFP or mCherry were seeded into a 24-well plate 1 day before SARS-CoV-2 infection. The infection was conducted as described above and images were captured at 32 h p.i. after staining with Hoechst 33342 for 15 min. Fluorescent images were acquired with an Eclipse Ti2 (Nikon, Tokyo, Japan) microscope, equipped with a PlanApo 20×/0.8 objective lens, a TI2-CTRE microscope controller (Nikon), a TI2-S-SE-E motorized stage (Nikon), and an X-Cite turbo system (Excelitas Technologies). The detector used in this study was a PRIME95B scientific complementary metal-oxide semiconductor (sCMOS) camera (Oxford Instruments). The sets of excitation and emission filters and dichroic mirrors adopted for this observation included GFP HQ (Nikon) for EGFP and Cy3 HQ (Nikon) for mCherry, and DAPI (Nikon) for Hoechst 33342. The colocalization area of the EGFP-expressing and mCherry-expressing cells and the number of nuclei in the syncytia were quantified with the use of the ‘measure colocalization’ or ‘count nuclei’ function of the MetaMorph software (Universal Imaging), respectively.

### Immunoblotting

Cells lysed on ice in lysis buffer [20 mM Tris-HCl (pH 7.4), 135 mM NaCl, 1% Triton-X 100, 10% glycerol] supplemented with a protease inhibitor cocktail, cOmplete mini (MilliporeSigma), were boiled in loading buffer and subjected to 5%–20% gradient SDS-PAGE. The proteins were transferred to polyvinylidene difluoride membranes (MilliporeSigma) and incubated with the anti-SARAS-CoV-2 S antibody (1:5,000 dilution; GeneTex), anti-N antibody (1:5,000 dilution; Sino Biological), or anti-GAPDH (1:5000 dilution; FUJIFILM Wako). The immune complexes were visualized with SuperSignal West Femto substrate (Thermo Fisher Scientific). The signals were detected using a WSE-LuminoGraph I (ATTO) and ImageSaver6 (ATTO).

### Airway-on-a-chip

Human lung microvascular endothelial cells (HMVEC-L) were obtained from Lonza (Cat# CC-2527) and cultured with EGM-2-MV medium (Lonza, Cat# CC-3202). The airway-on-a-chip were prepared as described previously^16^. In brief, the bottom channel of a polydimethylsiloxane (PDMS) device was pre-coated with fibronectin (3 μg/ml, Sigma-Aldrich, Cat# F1141). The microfluidic device was generated in accordance with our previous report^32^. HMVEC-L were suspended at 5 × 10^6^ cells/ml in the EGM2-MV medium. Then, 10 μl of suspension medium was injected into the fibronectin-coated bottom channel of the PDMS device. Next, the PDMS device was turned upside down and incubated for 1 h. After 1 h, the device was turned over and EGM2-MV medium was added into the bottom channel. After 4 days, human airway organoids (AO) were dissociated and seeded into the top channel. The AO were generated in accordance with our previous report^33^. The AO were dissociated into single cells and then suspended at 5 × 10^6^ cells/ml in AO differentiation medium. AO-derived airway epithelial cells were cultured in the top channel under an air-liquid interface condition for 5 days. Then, 10 μl of suspension medium was injected into the top channel. After 1 h, AO differentiation medium was added to the top channel. The cells were cultured under a humidified atmosphere with 5% CO_2_ at 37 °C. In the infection experiments, the AO differentiation medium containing either B.1.1, BA.1, BA.2, or BA.5 isolate (500 TCID_50_) was inoculated into the top channel. At 2 h.p.i., the top and bottom channels were washed and cultured with AO differentiation and EGM2-MV medium, respectively. The culture supernatants were collected, and viral RNA was quantified using RT–qPCR, and ratio of virus RNA amounts of the airway channel and blood vessel channel [Airway channel/ blood vessel channel] at 6 d.p.i. were calculated.

### Immunofluorescent staining

The PET membrane to which HMVEC-L were adhered was mechanically recovered from the uninfected and infected airway-on-a-chip and then used for immunofluorescent staining. The resulting cells were fixed with 4% paraformaldehyde and permeabilized with phosphate-buffered saline (PBS) containing 0.2% Triton X-100 and blocked with PBS containing 2% bovine serum albumin. The resulting cells were incubated with antibodies against VE-cadherin (Santa Cruz Biotechnology, Cat# sc-9989) followed by incubation with secondary antibodies conjugated with Alexa Fluor 594 (Thermo Fisher Scientific, Cat# A-11032). The cells were mounted with VECTASHIELD mounting medium with 4′,6-diamidino-2-phenylindole (DAPI) (Vector Laboratories, Cat# H-1200) and analyzed with a BZ-X700 (KEYENCE).

### FD-4 permeability tests

Top and bottom channels of airway-on-a-chip were rinsed with Hank’s Balanced Salt Solution (HBSS). The 300 μl of HBSS containing 25 μg/ml FD-4 (average molecular weight 3000–5000; Sigma-Aldrich) was added to the top channel, and 150 μl of HBSS was added to the bottom channel. After 70 min of incubation at 37 °C, the HBSS was collected from the bottom channel. The FD-4 fluorescent signal was measured with a fluorescence plate reader (TriStar 5; Berthold Technologies) using 490 nm excitation and 520 nm emission filters. FD-4 concentrations were calculated using the standard curve generated by serial dilution of FD-4.

The *Papp* in FD-4 permeability test was calculated according to the following equation.

$${Papp}={{{{{\rm{\delta }}}}}}{{{{{\rm{Cr}}}}}}/{{{{{\rm{\delta }}}}}}{{{{{\rm{t}}}}}}\times {{{{{\rm{Vr}}}}}}/({{{{{\rm{A}}}}}}\times {{{{{\rm{C}}}}}}0)$$

*δ*Cr/*δ*t = permeability rate (*δ*Cr = final concentration in the bottom channel, *δ*t = assay time); Vr = bottom channel volume; A = cell growth area; C0 = initial concentration in the top channel.

### RT-qPCR

RT-qPCR was performed as previously described^10,12^. Briefly, 5 μl of culture supernatant was mixed with 5 μl of 2× RNA lysis buffer [2% Triton X-100, 50 mM KCl, 100 mM Tris-HCl (pH 7.4), 40% glycerol, 0.8 U/μl recombinant RNase inhibitor (Takara-Bio)] and incubated at room temperature for 10 min. RNase-free water (90 μl) was added and the diluted sample (2.5 μl) was used as the template for real-time RT-PCR performed in accordance with the manufacturer’s protocol using the One Step TB Green PrimeScript PLUS RT-PCR kit (Takara-Bio) and the following primers: Forward *N*, 5′-AGC CTC TTC TCG TTC CTC ATC AC-3′; and Reverse *N*, 5′-CCG CCA TTG CCA GCC ATT C-3′. The viral RNA copy number was standardized with a SARS-CoV-2 direct detection RT-qPCR kit (Takara, Cat# RC300A). Fluorescent signals were acquired using QuantStudio Real-Time PCR system (Thermo Fisher Scientific), CFX Connect Real-Time PCR Detection system (Bio-Rad), Eco Real-Time PCR System (Illumina), qTOWER3 G Real-Time System (Analytik Jena), or 7500 Real-Time PCR System (Thermo Fisher Scientific).

To evaluate inflammation levels evoked by viral infection in hamsters, 500 μg of the lung RNA was used to synthesize cDNA with SuperScript IV VILO Master Mix. The resulting cDNA was used to quantify the expression of host genes^34,35^ (see Supplementary Table 1) with a Power SYBER Green Master Mix (Thermo Fisher Scientific) and QuantStudio Real-time PCR System (Thermo Fisher Scientific).

### Plasmid construction

The cDNA clones of EGFP and mCherry were inserted between the *Xho*I and *Xba*I sites of the lentiviral vector pCSII-EF-RfA^36^ using the infusion technique, and the resulting plasmids were designated pCSII-EF-EGFP and pCSII-EF-mCherry, respectively.

### Animal experiments

Syrian hamsters (male, 4 weeks old) were purchased from Japan SLC Inc. (Shizuoka, Japan). Baseline body weights, respiratory parameters, and SpO_2_ were measured before infection. For the virus infection experiments, hamsters were euthanized by intramuscular injection of a mixture of 0.15 mg/kg medetomidine hydrochloride (Domitor^®^, Nippon Zenyaku Kogyo), 2.0 mg/kg midazolam (Dormicum^®^, FUJIFILM Wako Chemicals), and 2.5 mg/kg butorphanol (Vetorphale^®^, Meiji Seika Pharma) or 0.15 mg/kg medetomidine hydrochloride, 2.0 mg/kg alphaxaone (Alfaxan®, Jurox), and 2.5 mg/kg butorphanol. The B.1.1 virus, Omicron subvariants (5 × 1000 TCID_50_ in 100 µl), and saline (100 µl) were intranasally inoculated under anesthesia. Oral swabs were collected at the indicated timepoints. Body weight was recorded daily by 7 d.p.i., and at 10 and 14 d.p.i. Enhanced pause (Penh, see below), the ratio of time to peak expiratory flow relative to the total expiratory time (Rpef, see below), and subcutaneous oxygen saturation (SpO_2_, see below) were monitored on 1, 3, 5, and 7 d.p.i. Lung tissues were collected at 2 and 5 d.p.i. Viral RNA load in the oral swabs and respiratory tissues was determined by RT-qPCR. Viral titers in the lung periphery were determined using TCID_50_. These tissues were also used for histopathological and IHC analyses (see below).

### Lung function test

Respiratory parameters (PenH and Rpef) were measured using a whole-body plethysmography system (DSI), in accordance with the manufacturer’s instructions. In brief, a hamster was placed in an unrestrained plethysmography chamber and allowed to acclimatize for 30 sec; then, data were acquired over a 2.5-min period using FinePointe Station and Review software v2.9.2.12849 (STARR). The state of oxygenation was examined by measuring SpO_2_ using a pulse oximeter, MouseOx PLUS (STARR). SpO_2_ was measured by attaching a measuring chip to the neck of hamsters sedated with 0.25 mg/kg medetomidine hydrochloride.

### H&E staining

H&E staining was performed as described in a previous report^10^. Briefly, excised animal tissues were fixed with 10% formalin neutral buffer solution and processed for paraffin embedding. The paraffin blocks were sectioned at a thickness of 3 µm and then mounted on silane-coated glass slides (MAS-GP, Matsunami). H&E staining was performed in line with a standard protocol.

### IHC

IHC was performed using an Autostainer Link 48 (Dako). The deparaffinized sections were exposed to EnVision FLEX target retrieval solution high pH (Agilent, Cat# K8004) for 20 min at 97 °C for activation, and mouse anti-SARS-CoV-2 N monoclonal antibody (R&D Systems, Clone 1035111, Cat# MAB10474-SP, 1:400) was used as a primary antibody. The sections were sensitized using EnVision FLEX (Agilent) for 15 min and visualized by peroxidase-based enzymatic reaction with 3,3′-diaminobenzidine tetrahydrochloride as a substrate for 5 min.

For the evaluation of N protein positivity in the tracheae at 2 d.p.i. and the lung specimens of infected hamsters at 2 and 5 d.p.i. (B1.1, BA.1, BA.2, and BA.5, *n* = 4 each), staining was performed with mouse anti-SARS-CoV-2 N monoclonal antibody (1:400). N-protein positivity was evaluated by certified pathologists as previously described^9,11^. Images were incorporated as virtual slides by NDP.scan software v3.2.4 (Hamamatsu Photonics). N-protein positivity was measured as the area using Fiji software v2.2.0 (ImageJ).

### Histopathological scoring of lung lesion

The inflamed area in the infected lungs was measured by the presence of type II pneumocyte hyperplasia. Four hamsters infected with each virus were sacrificed at 2 and 5 d.p.i., and all four lung lobes, including upper right (anterior/cranial), middle, lower (posterior/caudal), and accessory lobes, were sectioned along with their bronchi. For Immunohistochemistry (IHC) analyses, we prepared independent 72 slides in total including 36 lung specimens (16 of Day 2 as shown in Supplementary Fig. 5A and 20 of Day 5) and 36 tracheal specimens (16 of Day 2 as shown in Supplementary Fig. 4A and 20 of Day 5). Positivity of IHC was digitally analyzed by using Fiji software v2.2.0 (ImageJ). Three certified pathologists were blinded to evaluate each pathological feature of lungs such as bronchitis, hemorrhage, alveolar damage, Type II pneumocytes by using above 36 independent slides of H&E staining and scored shown as Fig. 4A and 4C. The tissue sections were stained by H&E, and the digital microscopic images were incorporated into virtual slides using NDRscan3.2 software (Hamamatsu Photonics). The color of the images was decomposed by RGB in split channels using Fiji software v2.2.0 showing in Supplementary Fig. 5A and Supplementary Fig. 6A.

Histopathological scoring was performed as described in a previous report with minor modifications. Briefly, pathological features including bronchitis or bronchiolitis, hemorrhage or congestion, alveolar damage with epithelial apoptosis and macrophage infiltration, hyperplasia of type II pneumocytes, and the area of the hyperplasia of large type II pneumocytes were evaluated by certified pathologists in the second most severe pulmonary lobe to properly reflect with whole pulmonary phenotype. The degree of these pathological findings was scored using a four-tiered system as follows: 0 (negative), 1 (weak), 2 (moderate), and 3 (severe). The "large type II pneumocytes" are hyperplasic type II pneumocytes with a nucleus of >10 μm in diameter. Total histology score is the sum of these five indices. In the representative lobe of each lung, the inflamed area with type II pneumocytes was gated by the certified pathologists on H&E staining, and the indicated area was measured by Fiji software v2.2.0.

### Viral genome sequencing analysis

The sequences of the working viruses were verified by viral RNA-sequencing analysis. Viral RNA was extracted using QIAamp viral RNA mini kit (Qiagen, Cat# 52906). The sequencing library for total RNA sequencing was prepared using NEB Next Ultra RNA Library Prep Kit for Illumina (New England Biolabs, Cat# E7530). Paired-end, 76-bp sequencing was performed using MiSeq (Illumina) with MiSeq reagent kit v3 (Illumina, Cat# MS-102-3001). Sequencing reads were trimmed using fastp v0.21.0^37^ and subsequently mapped to the viral genome sequences of a lineage B isolate (strain Wuhan-Hu-1; GISAID ID: EPI_ISL_402125; GenBank accession no. NC_045512.2) using BWA-MEM v0.7.17^38^. Variant calling, filtering, and annotation were performed using SAMtools v1.9^39^ and snpEff v5.0e^40^. Information on the detected mutations in the working virus stocks is summarized in Supplementary Table 2.

### RNA-Seq analysis

Total RNA was extracted from tissues using the procedure described above. The sequencing library was prepared using Illumina TruSeq Stranded mRNA Sample Preparation Kit (Illumina). Paired-end, 150-bp sequencing was performed using Illumina NovaSeq 6000 System (Illumina).

Sequencing reads were trimmed using fastp v0.21.0. The trimmed reads were subsequently mapped to the reference genome of Syrian hamsters (NCBI Accession: GCF_017639785.1) with the gene annotation file, both of which were downloaded from NCBI RefSeq (https://www.ncbi.nlm.nih.gov/refseq/), using STAR v 2.6.1c. The read count matrix was generated using featureCounts v1.6.3.

Of the hamster genes annotated by RefSeq, genes with orthologs in humans were analyzed in the present study. Information on the hamster-human ortholog relationship was extracted from the NCBI RefSeq database (https://www.ncbi.nlm.nih.gov/refseq/). Differential expression analysis was performed using DESeq2 v1.36.0. DEGs between infected and uninfected hamsters were determined using the Wald test, and DEGs among variants were determined using the likelihood ratio test. Genes with adjusted *P*-values calculated by the Benjamini–Hochberg (BH) method <0.05 and absolute values of log_2_ FC >1 were regarded as DEGs in the present study. Since the GO annotation information for hamster genes (GCF_017639785.1) was not available, we transferred the GO annotation information of human genes to orthologous hamster genes. GO enrichment analysis was performed using Fisher’s exact test. GO terms with adjusted *P*-value calculated by the BH method <0.1 were regarded as significant terms. The source data is available in Supplementary Data 1, 2.

### Statistics and reproducibility

Viral RNA copy, body weight, PenH, Rpef, and SpO_2_, and inflammatory mRNA gene levels obtained from the in vivo experiments were analyzed by repeated measures analysis of variance. Inflammation measures upon infection in vivo, the mRNA of the lung hilum and periphery areas at 2 d.p.i., and four host genes (*Cxcl10*, *Il-6*, *Isg15*, and *Mx-1*) were compared among Omicron subvariants using analysis of variance. Regarding PenH, Rpef, and SpO_2_, we compared infected animals with each variant against uninfected animals and calculated *p*-values using Dunnett’s adjustment. The other measurements were tested by Tukey’s multiplicity correction to maintain the type I error rate for comparison among infected or uninfected animals. These analyses were conducted using SAS Ver. 9.4 (SAS Institute, Cary, NC). The statistical significance of differences between BA.5 and other variants or saline across timepoints from day 1 p.i. to day 7 p.i. was tested using the Holm method. The indicated analyses were performed in R v4.1.2 (R Core Team, Vienna, Austria). The two-sided significance level was set to 0.05.

### Reporting summary

Further information on research design is available in the Nature Portfolio Reporting Summary linked to this article.

## Supplementary information

Supplementary Information

Description of Additional Supplementary Files

Supplementary Data 1

Supplementary Data 2

Supplementary Data 3

Reporting Summary

## Data Availability

The raw data of RNA-Seq are available on Sequence Read Archive (https://www.ncbi.nlm.nih.gov/sra; Accession PRJDB14143). The source data behind the graphs in the figures is available in Supplementary Data 3. The raw data of immunoblotting is deposited in Supplementary Fig. 9.

## Code availability

Computational codes used in the present study are available on the GitHub repository (https://github.com/TheSatoLab/Omicron_BA1_BA2_BA5_comparision).
