# Supplementary material for: Comparative pathogenicity of SARS-CoV-2 Omicron subvariants including BA.1, BA.2, and BA.5
Source: Commun Biol. 2023 Jul 24;6:772. doi: 10.1038/s42003-023-05081-w (PMC10366110; doi:10.1038/s42003-023-05081-w)
Supplement: Supplementary file 1 — Supplementary Information [file 42003_2023_5081_MOESM1_ESM.pdf]

Tamura et al. Supplementary Fig. 1

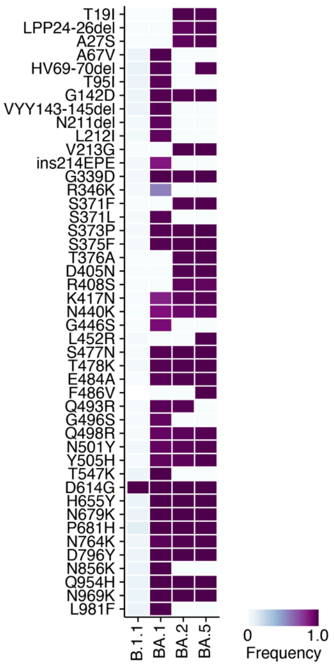

Supplementary Fig. 1. Profile of S substitutions among the Omicron subvariants used in this study. Heatmap representing the profile of S substitutions of B.1.1, BA.1, BA.2, and BA.5. Substitutions detected in ≥50% sequences of any lineages are summarized.

Tamura et al. Supplementary Fig. 2

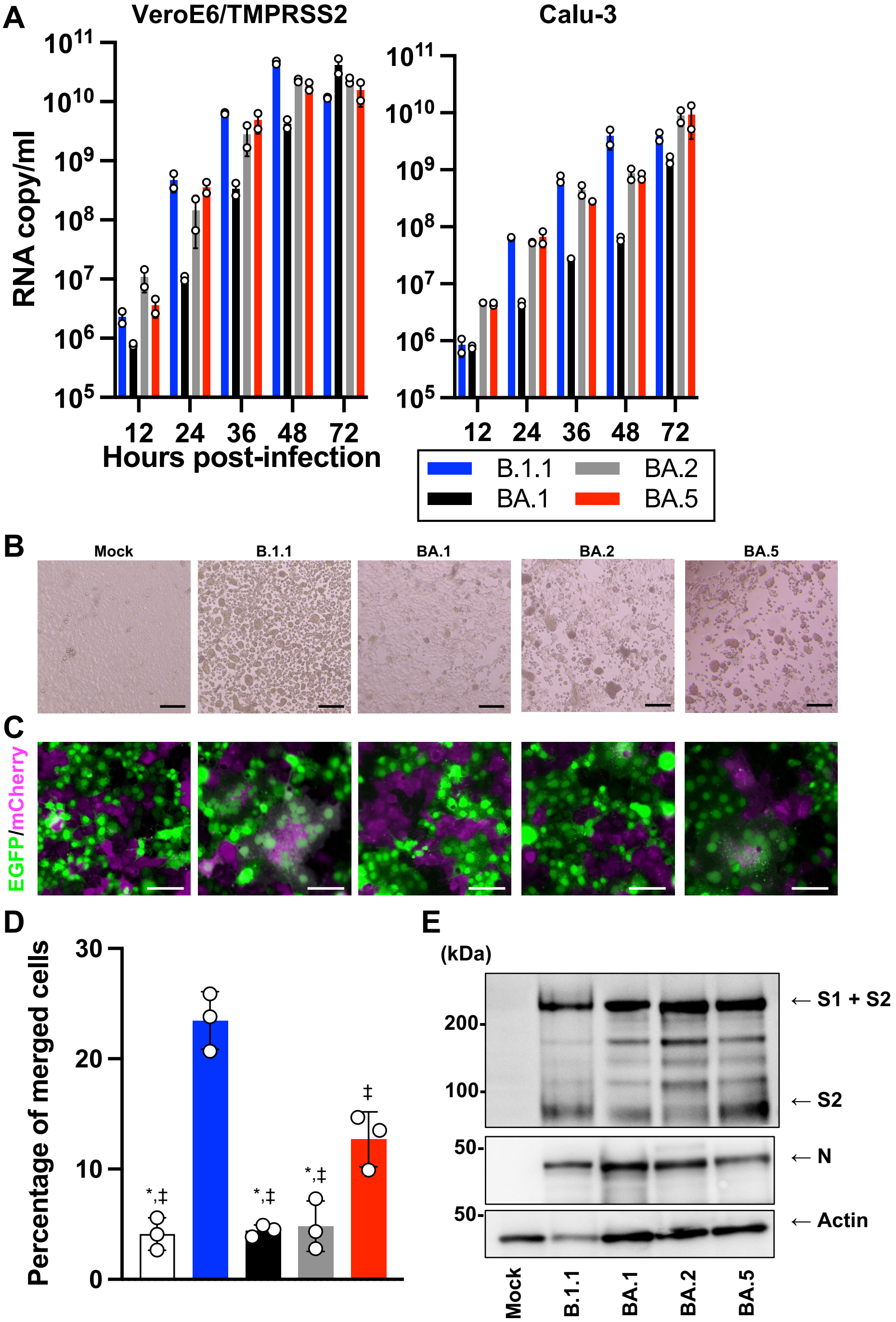

Supplementary Fig. 2. Virological features of Omicron subvariants *in vitro*, related to Fig.1. (**A**) *In vitro* growth kinetics of the four clinical isolates of B.1.1, Omicron BA.1, BA.2, and BA.5 were shown. The viral RNA copies in the supernatants were quantified by qRT-PCR. (**B**) Bright-field images of infected VeroE6/TMPRSS2 cells (m.o.i. = 0.01) at 32 h.p.i. (**C**) SARS-CoV-2 induced syncytial formation. EGFP- and mCherry-expressing VeroE6/TMPRSS2 cells were co-cultured at a 1:1 ratio and infected with B.1.1, BA.1, BA.2, and BA.5 isolates. Scale bars, 200 µm. Syncytial formation was monitored by immunofluorescent microscopy at 32 h.p.i. Scale bars, 100 µm. The representative images are shown. (**D**) Percentage of syncytia displayed as both EGFP and mCherry positive cells were calculated and shown as bar graphs. Statistically significant differences between B1.1 and other variants (‡, $P < 0.05$), between BA.5 and other variants (*, $P < 0.05$), and between BA.1 and other variants (†, $P < 0.05$) were determined by Tukey's multiplicity correction. (**E**) Expression of S protein (S1+S2 and S2) was examined by immunoblotting using monoclonal antibody against S upon infection with the respective SARS-CoV-2 variants in the VeroE6/TMPRSS2 cells.

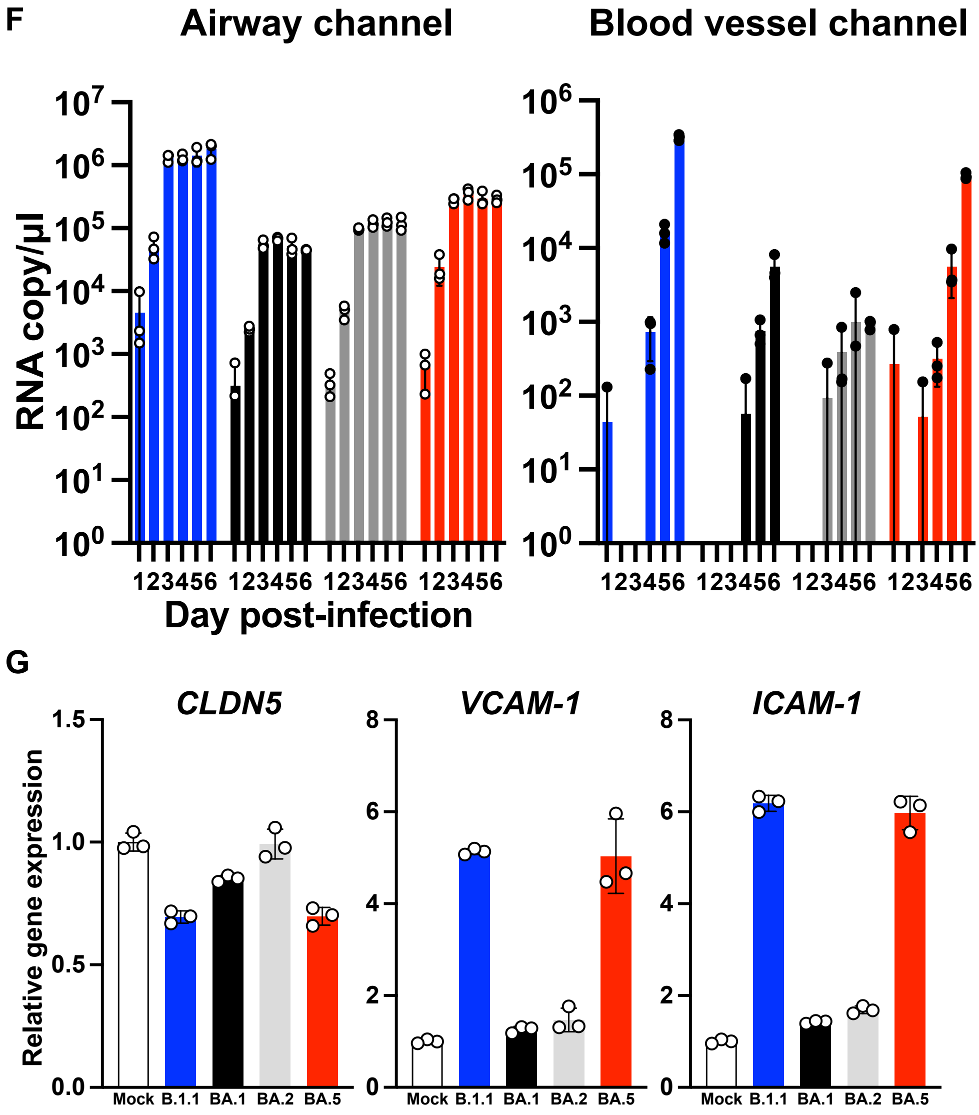

Supplementary Fig. 2. Virological features of Omicron subvariants *in vitro*, related to Fig.1. (**F**) Airway-on-a-chip analysis. Viral RNA in the supernatant of both airway and blood vessel channels was shown in bar graph. Data are the mean ± s.d. (**G**) The gene expression levels of *CLDN5*, *VCAM-1*, and *ICAM-1* in uninfected and infected endothelial cells of airway-on-a-chip. Mock = 1. Data are the mean ± s.d. Statistically significant differences between mock and the variants (*, P < 0.05) were determined by One-way ANOVA followed by Dunnett's post hoc test. Assays were performed independently in duplicate (**A**) or triplicate (**B**,**D**,**F**,**G**). In **B** and **C**, the representative images are shown.

Tamura et al. Supplementary Fig. 3

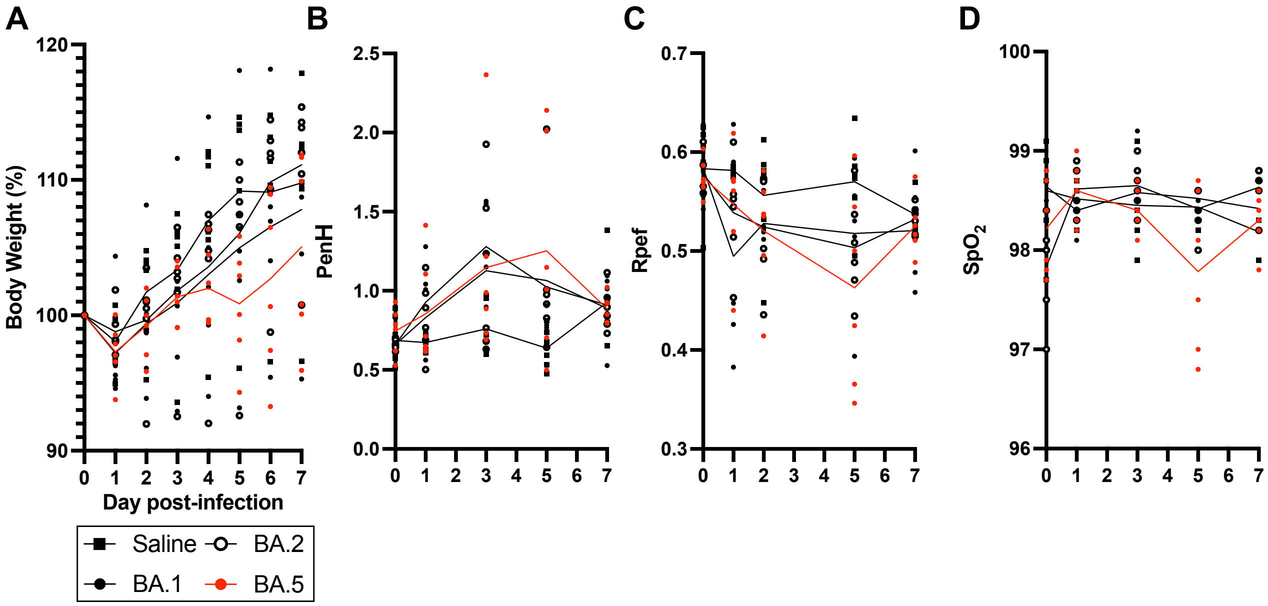

Supplementary Fig. 3. *In vivo* dynamics of Omicron subvariants, related to Fig. 2. Syrian hamsters were intranasally inoculated with BA.1 (n = 4), BA.2 (n = 4), and BA.5 (n = 4). Dynamics of body weight (**A**), PenH (**B**), Rpef (**C**), and SpO_2 (**D**) among Omicron subvariants by 7 d.p.i. are shown. Saline injection served as a control in this study.

Tamura et al. Supplementary Fig. 4

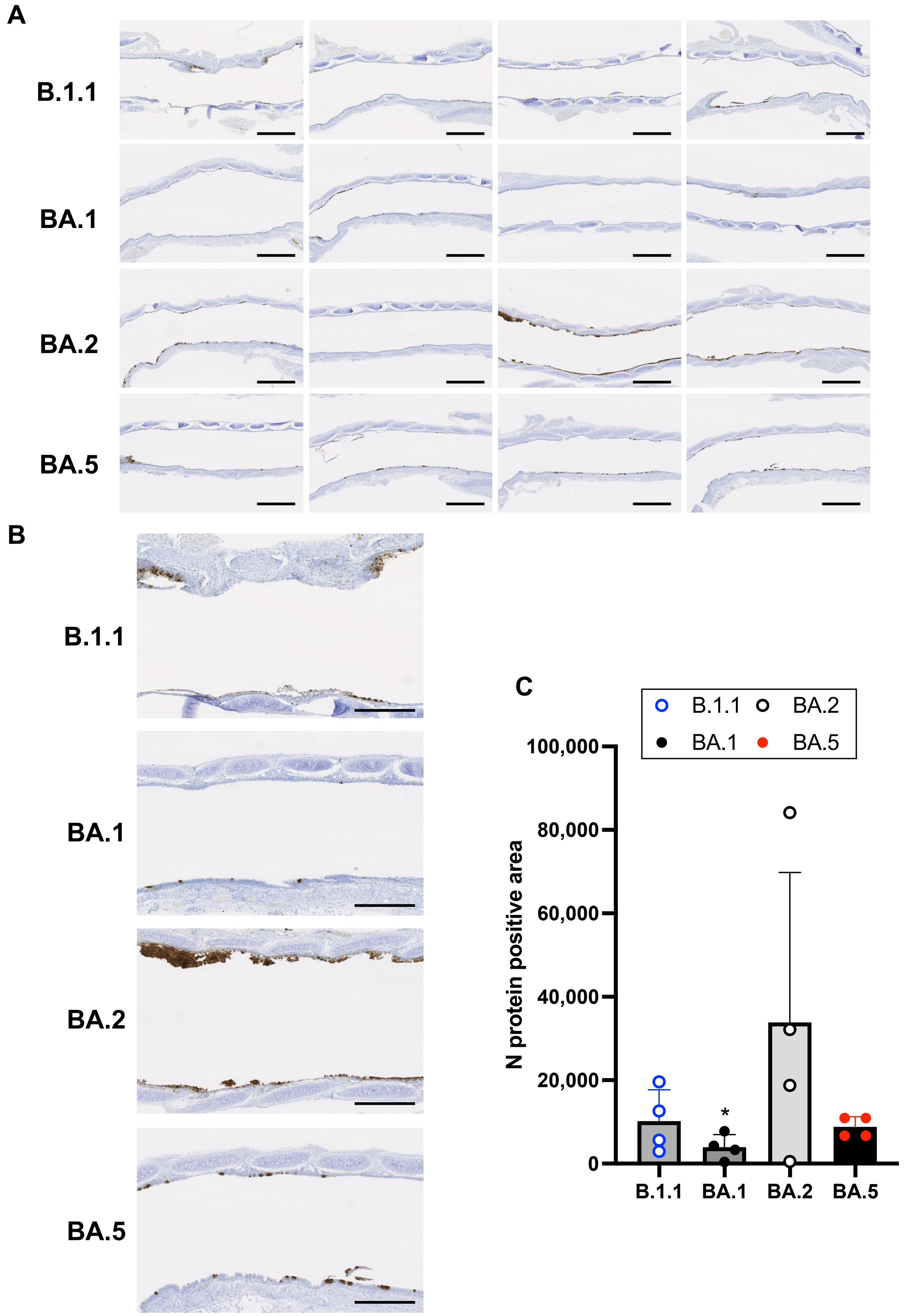

Supplementary Fig. 4. Virological features of Omicron subvariants *in vivo* in the trachea, related to Fig. 3. (A) Percentage of N-positive cells in the upper part of the trachea from the oral entrance at the vertical levels of thyroid cartilage at 2 d.p.i. is shown. Scale bars, 1 mm. (**B**) Representative images for each virus. Brown color indicates N-positive cells. Scale bars, 500 μm. (**C**) The infected area of N-positive cells was digitized using ImageJ and displayed in graph. In **C**, data are the mean ± s.e.m. and each dot indicates the result from an individual hamster.The statistical significance of differences between BA.5 and other variants (*, $P < 0.05$) by Tukey's multiplicity correction.

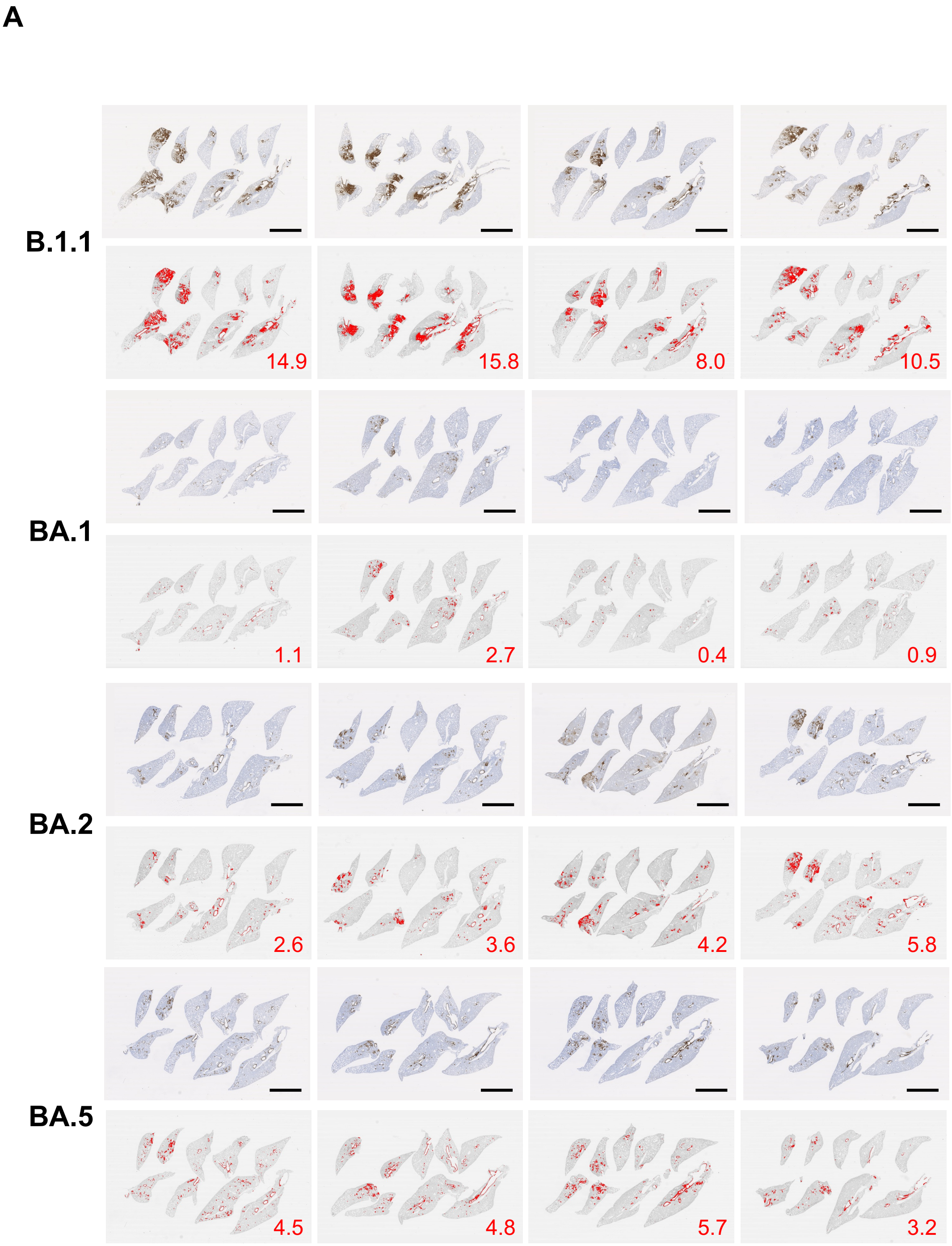

Supplementary Fig. 5. Virological features of Omicron subvariants *in vivo* in the lung, related to Fig. 3. (**A**) Percentage of N-positive cells in whole lung lobes at 2 d.p.i. is shown. Scale bars, 5 mm.

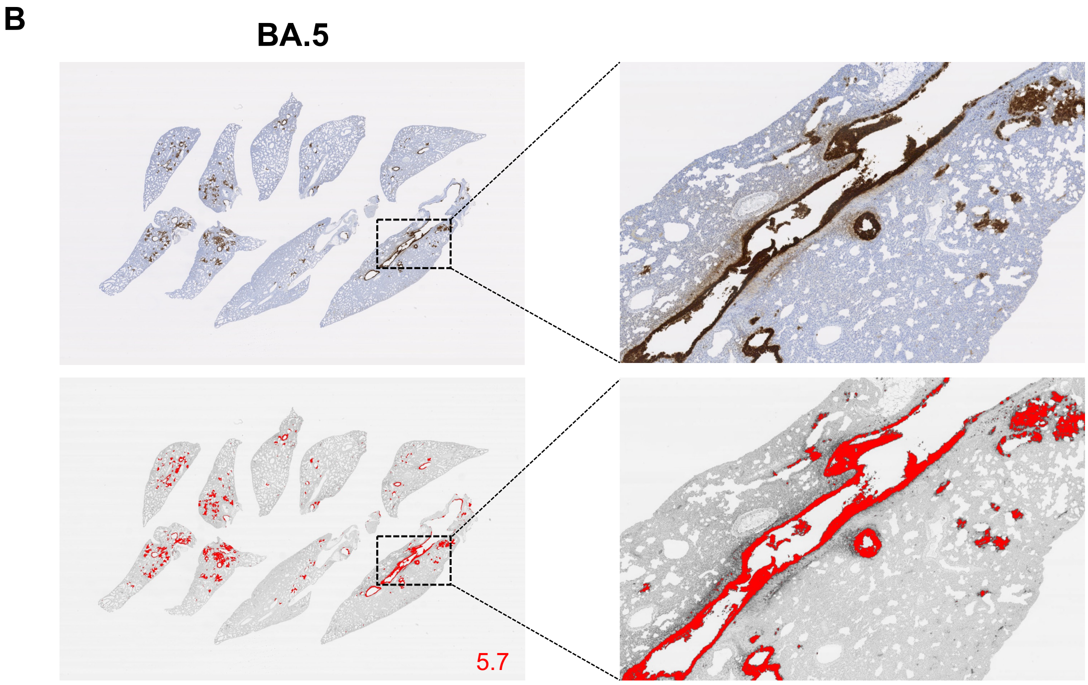

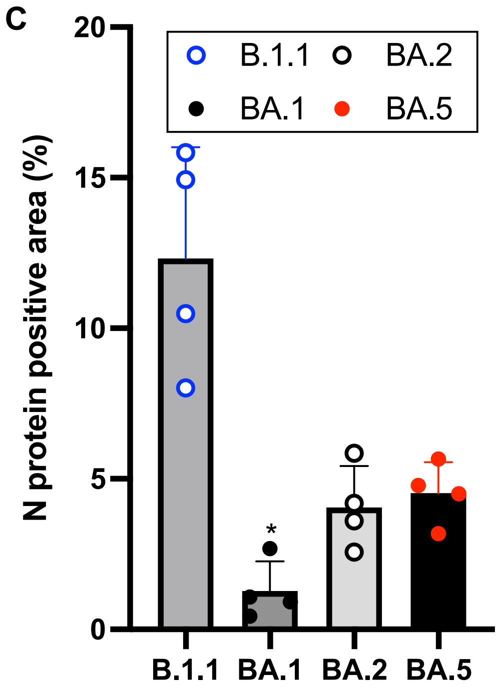

Supplementary Fig. 5. Virological features of Omicron subvariants *in vivo* in the lung, related to Fig. 3. (**B**) Enlarged images of N-positive area in BA.5-infected lung. The N-positive areas shown in brown were digitized using ImageJ (red color). (**C**) Percentage of the N-positive area at 2 d.p.i. is shown in graph. In **C**, data are the mean ± s.e.m. and each dot indicates the result from an individual hamster. In **C**, data are the mean ± s.e.m. and each dot indicates the result from an individual hamster.The statistical significance of differences between BA.5 and other variants (*, P < 0.05) by Tukey's multiplicity correction.

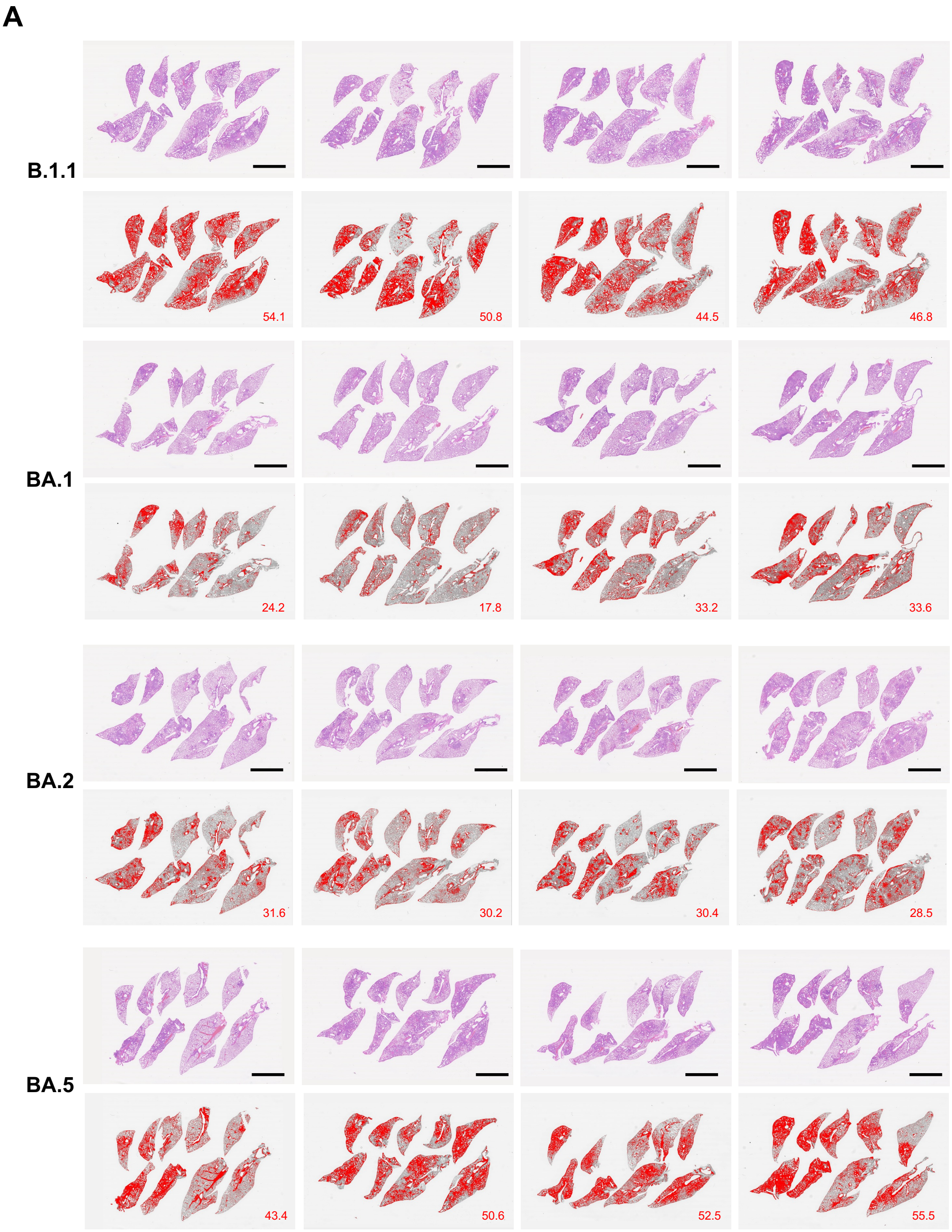

Supplementary Fig. 6. Pathological features of Omicron subvariants *in vivo*, related to Fig. 4. (**A**) Sections of all four lung lobes at 5 d.p.i. H&E staining and the inflammatory area with type II pneumocytes are shown. The inflammatory area is colored red. The number in the panel indicates the percentage of the section represented by the indicated area. Scale bars, 5 mm.

Tamura et al. Supplementary Fig. 6

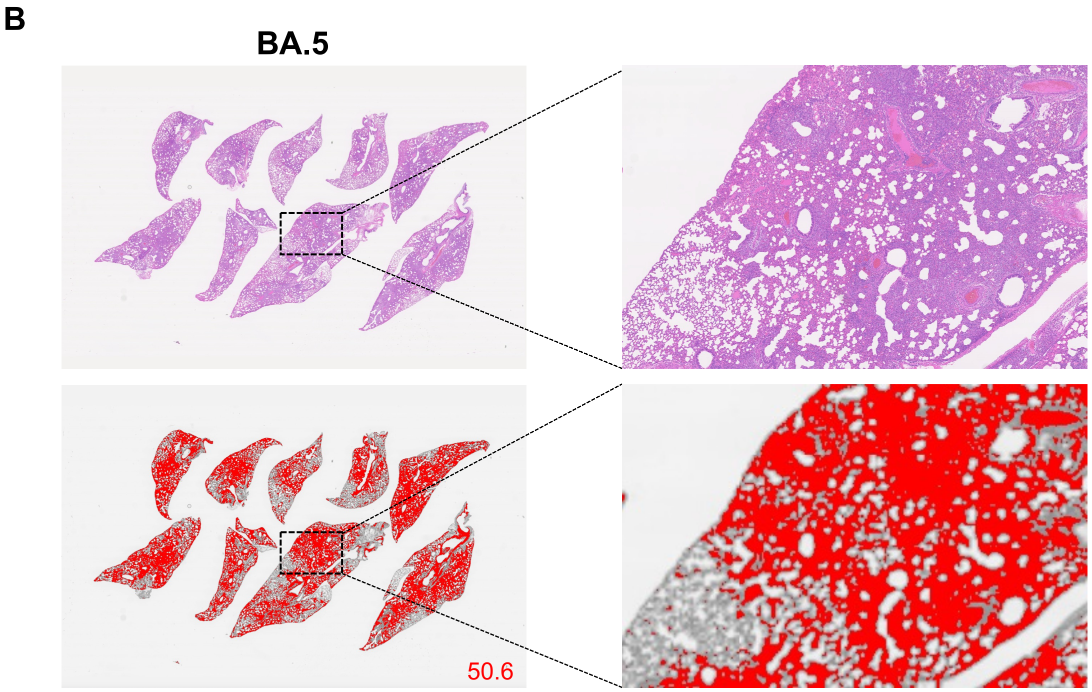

Supplementary Fig. 6. Pathological features of Omicron subvariants *in vivo*, related to Fig. 4. (**B**) Enlarged images of the inflammatory area in BA.5-infected lung. The inflammatory area with type II pneumocytes were digitized using ImageJ (red color) and shown in graph (**Fig. 4C**).

Tamura et al. Supplementary Fig. 7

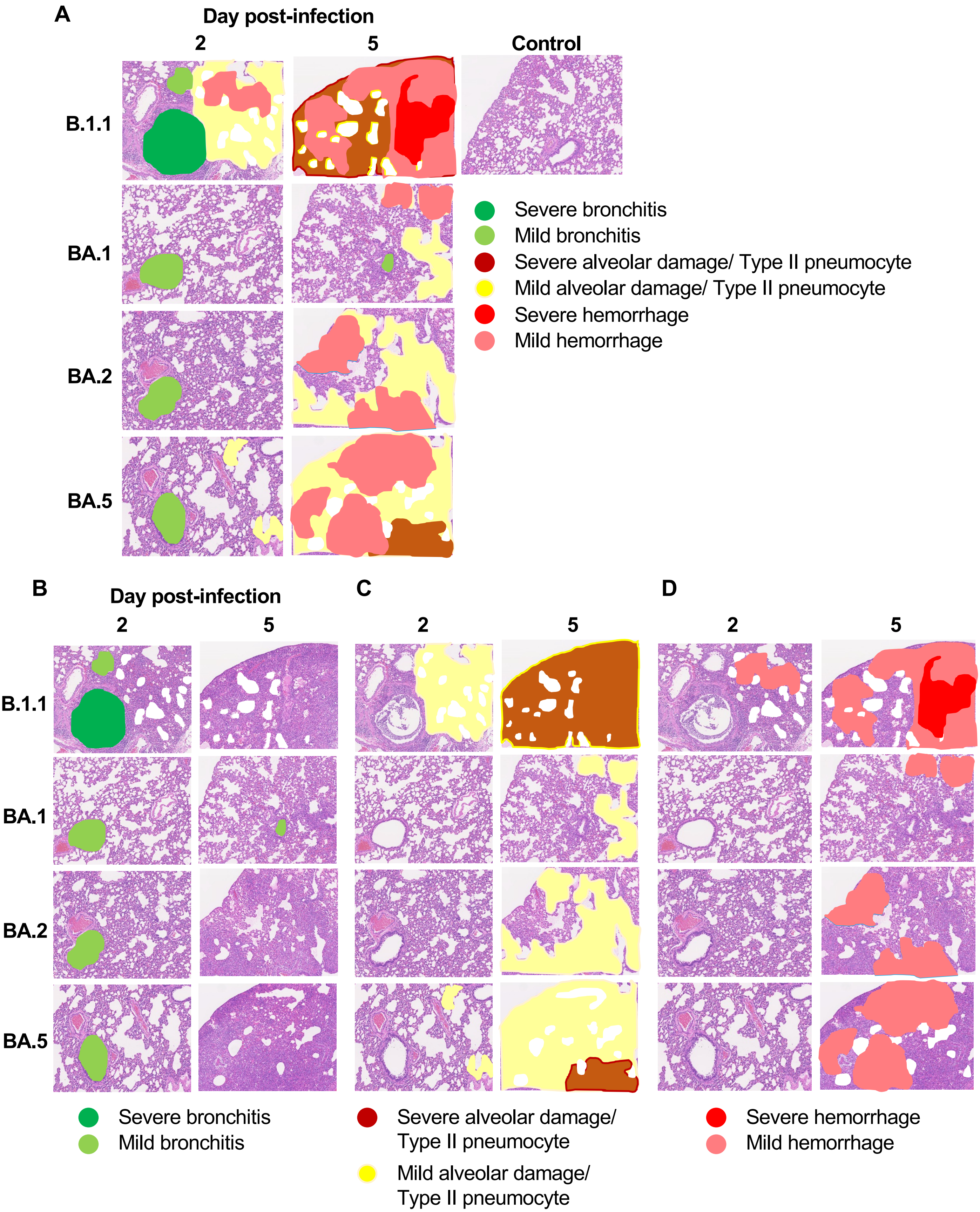

Supplementary Fig. 7. Graphical representation of pathological phenotypes, related to Fig. 4. (**A**) Area of bronchitis, alveolar damage, Type II pneumocyte, and hemorrhage shown in Fig. 4A are mapped in representative photographs of Fig. 4B. **B-D.** Individual phenotype such as bronchitis (**B**), alveolar damage/Type II pneumocyte (**C**), and hemorrhage (**D**) are shown along with their strengths and weakness.

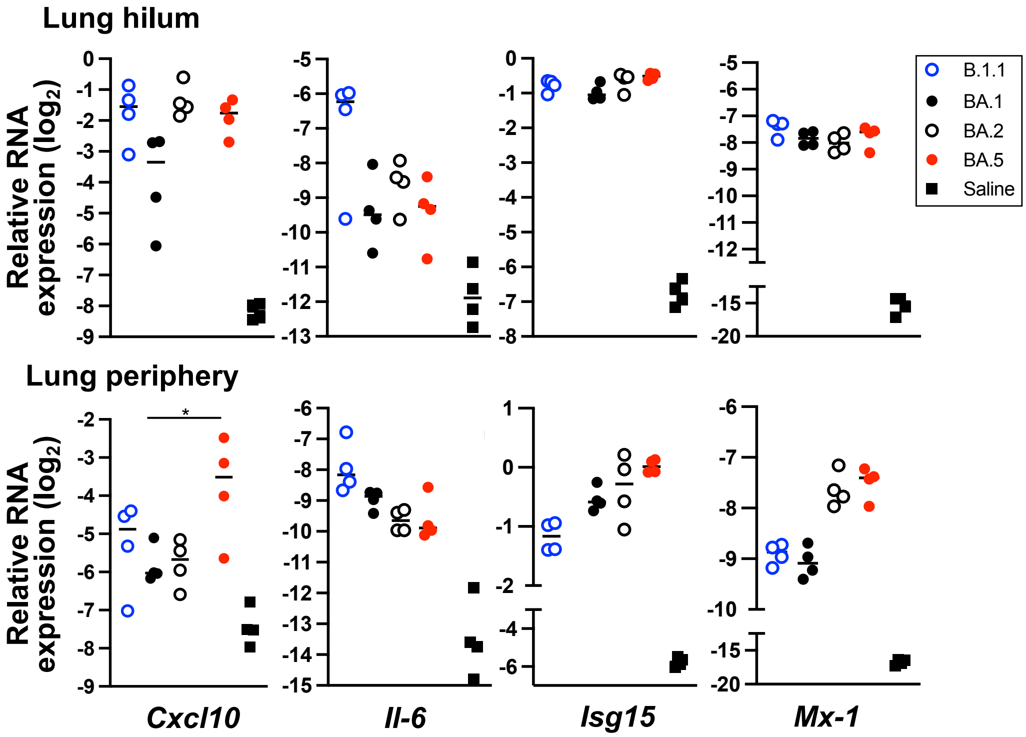

Supplementary Fig. 8. Gene expression alteration of hamster lungs upon infection with Omicron subvariants related to Fig. 5. mRNA of the lung tissues obtained at day 2 post-infection was used to measure expression levels of inflammatory genes (*Cxcl10*, *Il-6*, *Isg15*, and *Mx-1*) with normalization using the housekeeping gene *B-actin*. Each dot indicates the result from an individual hamster. The statistical significance of differences among Omicron subvariants was tested by Tukey's multiplicity correction (*, $P < 0.05$).

A

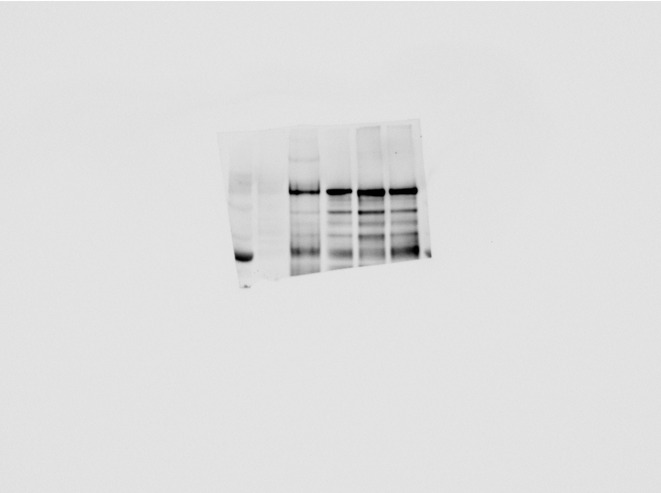

B

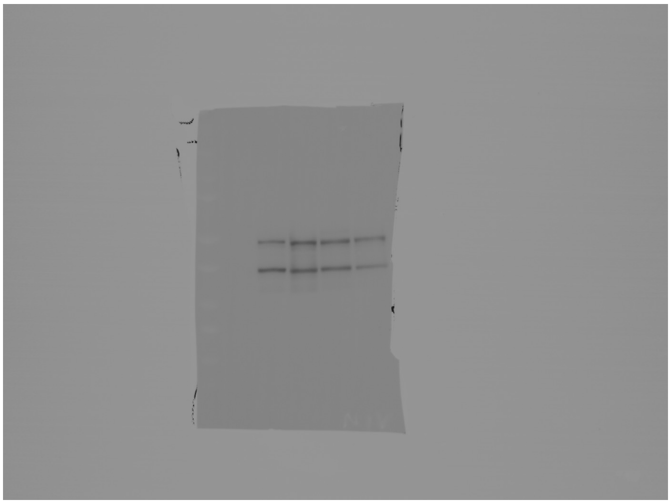

C

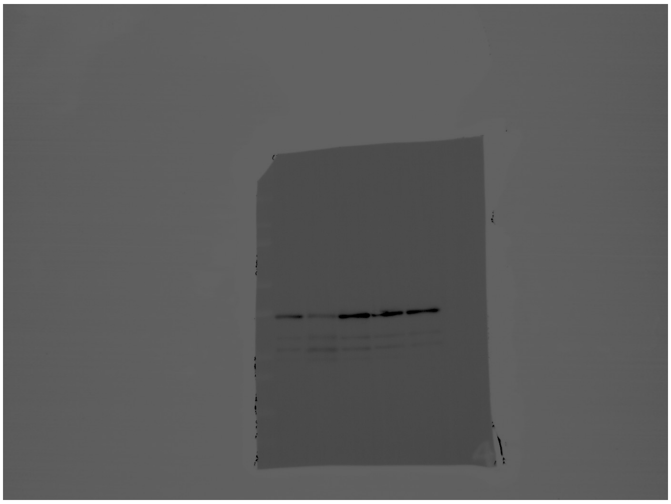

Supplementary Fig. 9. Unedited gel images related to Fig. S2E. (**A**) Raw image of blotting by anti-S protein antibody. (**B**) Raw image by anti-N protein antibody. (**C**) Raw image by anti-beta actin antibody.

Supplementary Table 1. Primers used for quantifying the host genes of hamster upon infection with the SARS-CoV-2 variants in this study.

Host gene	Forward primer	Reverse primer
CXCL10	GCCATTCATCCACAGTTGACA	CATGGTGCTGACAGTGGAGTCT
IL-6	CCTGAAAGCACTTGAAGAATTCC	GGTATGCTAAGGCACAGCACACT
ISG15	AAAGCCTACAGCCATGACCT	TTAGTCAGGGGCACCAGGAA
MX-1	GCGCTTCCAGACTCTTCTGA	CCTAAGATACATGCGATGGCG
RPL18	GTTTATGAGTCGCACTAACCG	TGTTCTCTCGGCCAGGAA
BACTIN	ACTGCCGCATCCTCTTCCT	TCGTTGCCAATGGTGATGAC

Supplementary Table 2. Mutations in Omicron subvariants.

Strain name[a]	Position[b]	REF	ALT	Quality score	Depth in REF	Depth in ALT	Variant type	Gene	Amino acid change
B.1.1 (EPI_ISL_479681)	14408	C	T	225	0	190	missense_variant	NSP12	P303L
BA.1 (EPI_ISL_7418017)	10448	CC	TA	225	0	218	missense_variant	NSP5	P132Y
	11750	C	T	159	177	56	missense_variant	NSP6	L260F
	20486	A	C	222	76	86	missense_variant	NSP15	K289T
BA.5 (EPI_ISL_12812500)	2388	C	T	228	2	227	missense_variant	NSP2	T528I
	11750	C	T	222	140	104	missense_variant	NSP6	L260F

[a] No mutation was detected in BA.2 (PI_ISL_9595859).

[b] Refer the amino acid sequence of SARS-CoV-2 Wuhan-Hu-1 (NC_045512.2)
