# Supplementary material for: Comparative pathogenicity of SARS-CoV-2 Omicron subvariants including BA.1, BA.2, and BA.5
Source: Commun Biol. 2023 Jul 24;6:772. doi: 10.1038/s42003-023-05081-w (PMC10366110; doi:10.1038/s42003-023-05081-w)
Supplement: Supplementary file 2 — Description of Additional Supplementary Files [file 42003_2023_5081_MOESM2_ESM.pdf]

Description of Additional Supplementary Files

File name: Supplementary Data 1
Description: Results of clustering analysis, related to Fig. 5C

File name: Supplementary Data 2
Description: Results of GO enrichment analysis, related to Fig. 5D

File name: Supplementary Data 3
Description: Source data behind the graphs in the figures.
