# Supplementary material for: Comparative pathogenicity of SARS-CoV-2 Omicron subvariants including BA.1, BA.2, and BA.5
Source: Commun Biol. 2023 Jul 24;6:772. doi: 10.1038/s42003-023-05081-w (PMC10366110; doi:10.1038/s42003-023-05081-w)
Supplement: Supplementary file 6 — Reporting Summary [file 42003_2023_5081_MOESM6_ESM.pdf]

nature portfolio

Corresponding author(s): Isao Yokota, Keita Matsuno, Kazuo Takayama, Shinya Tanaka, Kei Sato, Takasuke Fukuhara

Last updated by author(s): Jun 9, 2023

Reporting Summary

Nature Portfolio wishes to improve the reproducibility of the work that we publish. This form provides structure for consistency and transparency in reporting. For further information on Nature Portfolio policies, see our Editorial Policies and the Editorial Policy Checklist.

Statistics

For all statistical analyses, confirm that the following items are present in the figure legend, table legend, main text, or Methods section.

n/a	Confirmed	
☐	☒	The exact sample size (*n*) for each experimental group/condition, given as a discrete number and unit of measurement
☐	☒	A statement on whether measurements were taken from distinct samples or whether the same sample was measured repeatedly
☐	☒	The statistical test(s) used AND whether they are one- or two-sided<br>*Only common tests should be described solely by name; describe more complex techniques in the Methods section.*
☒	☐	A description of all covariates tested
☒	☐	A description of any assumptions or corrections, such as tests of normality and adjustment for multiple comparisons
☐	☒	A full description of the statistical parameters including central tendency (e.g. means) or other basic estimates (e.g. regression coefficient) AND variation (e.g. standard deviation) or associated estimates of uncertainty (e.g. confidence intervals)
☐	☒	For null hypothesis testing, the test statistic (e.g. *F*, *t*, *r*) with confidence intervals, effect sizes, degrees of freedom and *P* value noted<br>*Give P values as exact values whenever suitable.*
☐	☒	For Bayesian analysis, information on the choice of priors and Markov chain Monte Carlo settings
☒	☐	For hierarchical and complex designs, identification of the appropriate level for tests and full reporting of outcomes
☒	☐	Estimates of effect sizes (e.g. Cohen's *d*, Pearson's *r*), indicating how they were calculated

Our web collection on statistics for biologists contains articles on many of the points above.

Software and code

Policy information about availability of computer code

Data collection	QuantStudio 1 Real-Time PCR system (Thermo Fisher Scientific)<br>QuantStudio 3 Real-Time PCR system (Thermo Fisher Scientific)<br>QuantStudio 5 Real-Time PCR system (Thermo Fisher Scientific)<br>Eclipse Ts2 microscope (Nikon)<br>TI2-CTRE microscope controller (Nikon)<br>TI2-S-SE-E motorized stage (Nikon)<br>X-Cite turbo system (Excelitas Technologies)PRIME95B scientific complementary metal-oxide semiconductor (sCMOS) camera (Oxford Instruments)<br>GFP HQ (Nikon)<br>Cy3 HQ (Nikon)<br>WSE-LuminoGraph I (ATTO)<br>BZ-X700 (KEYENCE)<br>JEM-1400 (JEOL)<br>whole-body plethysmography system (DSI)<br>Illumina NovaSeq 6000 System (Illumina)
Data analysis	Sequencher software v5.1 (Gene Codes Corporation)<br>Excel software v16.16.8 (Microsoft)<br>Prism 9 software v9.1.1 (GraphPad Software)<br>Metamorph software (Universal Imaging)<br>Fiji software v2.2.0 (ImageJ)

ImageSaver6 (ATTO)
FinePointe Station and Review software v2.9.2.12849 (STARR)
Pulse oximeter, MouseOx PLUS (STARR)
SAS Ver. 9.4 (SAS Institute, Cary, NC)
R v4.1.2 (R Core Team, Vienna, Austria)

For manuscripts utilizing custom algorithms or software that are central to the research but not yet described in published literature, software must be made available to editors and reviewers. We strongly encourage code deposition in a community repository (e.g. GitHub). See the Nature Portfolio guidelines for submitting code & software for further information.

Data

Policy information about availability of data

All manuscripts must include a data availability statement. This statement should provide the following information, where applicable:

- Accession codes, unique identifiers, or web links for publicly available datasets
- A description of any restrictions on data availability
- For clinical datasets or third party data, please ensure that the statement adheres to our policy

The raw data of RNA-Seq are available on Sequence Read Archive (https://www.ncbi.nlm.nih.gov/sra; Accession PRJDB14143). Computational codes used in the present study are available on the GitHub repository (https://github.com/TheSatoLab/Omicron_BA1_BA2_BA5_comparision).

Human research participants

Policy information about studies involving human research participants and Sex and Gender in Research.

Reporting on sex and gender	N/A
Population characteristics	N/A
Recruitment	N/A
Ethics oversight	N/A

Note that full information on the approval of the study protocol must also be provided in the manuscript.

Field-specific reporting

Please select the one below that is the best fit for your research. If you are not sure, read the appropriate sections before making your selection.

☒ Life sciences ☐ Behavioural & social sciences ☐ Ecological, evolutionary & environmental sciences

For a reference copy of the document with all sections, see nature.com/documents/nr-reporting-summary-flat.pdf

Life sciences study design

All studies must disclose on these points even when the disclosure is negative.

Sample size	The sample sizes (n > 3) for cell culture experiments were chosen for applying statistical tests. The sample sizes (n > 3) for the hamster studies were chosen because they have previously been shown to be sufficient to evaluate a significant difference among groups (Belser et al., Nature, 2013; Zhang et al., Science, 2013; Imai et al., Nature Microbiology, 2020; Saito et al., Nature, 2021).
Data exclusions	N/A
Replication	In vitro experiments representative of at least 2 experiments with multiple samples per time point. In vivo experiments (hamster) utilized multiple animals per group per time point and were from more than single experiment. In vivo experiments were replicated and performed independently. All attempts at replication were successful.
Randomization	No method of randomization was used to determine how the animals were allocated to the experimental groups and processed in this study, because covariates (sex and age) were identical (male, 4 weeks old). For experiments other than animal studies, randomization is not applicable because homogenous materials (i.e., cell lines) were used. Therefore, randomization is not applicable.
Blinding	No blinding was carried out, because these are not relevant for an observational study.

Reporting for specific materials, systems and methods

We require information from authors about some types of materials, experimental systems and methods used in many studies. Here, indicate whether each material, system or method listed is relevant to your study. If you are not sure if a list item applies to your research, read the appropriate section before selecting a response.

Materials & experimental systems

n/a	Involved in the study
☐	☒ Antibodies
☐	☒ Eukaryotic cell lines
☒	☐ Palaeontology and archaeology
☐	☒ Animals and other organisms
☒	☐ Clinical data
☒	☐ Dual use research of concern

Methods

n/a	Involved in the study
☒	☐ ChIP-seq
☒	☐ Flow cytometry
☒	☐ MRI-based neuroimaging

Antibodies

Antibodies used	For IHC:<br>mouse anti-SARS-CoV-2 N monoclonal antibody (R&D systems, Clone 1035111, Cat# MAB10474-SP, 1:400)<br>For Western blot:<br>anti-SARAS-CoV-2 S antibody (GeneTex, Clone 1A9, Cat# GTX632604, 1:5,000)<br>anti-SARS-CoV-2 N antibody (Sino Biological, Clone 05, Cat# 40143-MM05, 1:5,000)<br>anti-GAPDH (FUJIFILM Wako, Clone 5A12, 1:5,000)
Validation	Validation was conducted by manufacturers prior to sale, and validation statements are available on the manufacturers' website.

Eukaryotic cell lines

Policy information about cell lines and Sex and Gender in Research

Cell line source(s)	HEK293-ACE2 cells (HEK293 cells stably expressing human ACE2) (Motozono et al., Cell Host & Microbe, 2021)<br>VeroE6/TMPRSS2 cells (JCRB1819) (Matsuyama et al., Proc Natl Acad Sci, 2020)<br>Calu-3 cells (a human lung epithelial cell line; ATCC HTB-55)<br>iPSC-derived alveolar epithelial cells (Yamamoto et al., Nat Methods, 2017)<br>Airway-on-a-chip composed of human lung microvascular endothelial cells and airway organoids (Hashimoto et al., Sci Adv, 2022)
Authentication	None of the cells used were authenticated.
Mycoplasma contamination	All cell lines were regularly tested for mycoplasma contamination by using PCR and were confirmed to be mycoplasma-free.
Commonly misidentified lines<br>(See ICLAC register)	No commonly misidentified cell lines were used.

Animals and other research organisms

Policy information about studies involving animals; ARRIVE guidelines recommended for reporting animal research, and Sex and Gender in Research

Laboratory animals	Syrian hamsters (male, 4 weeks old) were purchased from Japan SLC Inc. (Shizuoka, Japan).
Wild animals	No wild animal was used in this study.
Reporting on sex	Epidemiological studies of the COVID-19 patients have suggested the male bias in outcomes of lung illness. In addition, hamster model, male hamsters have been reported to be more susceptible to SARS-CoV-2 infection (Lunzhi Yuan et al Signal Transduction and Targeted study, 2021). Therefore, also in this study, male hamsters were used.
Field-collected samples	No field collected sample was used in the study.
Ethics oversight	All experiments with hamsters were performed in accordance with the Science Council of Japan's Guidelines for the Proper Conduct of Animal Experiments. The protocols were approved by the Institutional Animal Care and Use Committee of National University Corporation Hokkaido University (approval numbers 20-0123 and 20-0060).

Note that full information on the approval of the study protocol must also be provided in the manuscript.
